# Hyperaminoacidemia induces pancreatic α cell proliferation via synergism between the mTORC1 and CaSR-Gq signaling pathways

**DOI:** 10.1038/s41467-022-35705-4

**Published:** 2023-01-16

**Authors:** Yulong Gong, Bingyuan Yang, Dingdong Zhang, Yue Zhang, Zihan Tang, Liu Yang, Katie C. Coate, Linlin Yin, Brittney A. Covington, Ravi S. Patel, Walter A. Siv, Katelyn Sellick, Matthew Shou, Wenhan Chang, E. Danielle Dean, Alvin C. Powers, Wenbiao Chen

**Affiliations:** 1 Department of Molecular Physiology & Biophysics, Vanderbilt University, 2215 Garland Ave, Nashville, TN 37232 USA; 2 State Key Laboratory of Freshwater Ecology and Biotechnology, Institute of Hydrobiology, Chinese Academy of Sciences, Wuhan, Hubei 430072 China; 3 College of Animal Science and Technology, Nanjing Agricultural University, Nanjing, 210095 China; 4 Division of Diabetes, Endocrinology and Metabolism, Department of Medicine, Vanderbilt University Medical Center, 2215 Garland Ave, Nashville, TN 37232 USA; 5 University of California San Francisco and San Francisco VA Medical Center, San Francisco, CA 94158 USA; 6 VA Tennessee Valley Healthcare System, Nashville, TN 37212 USA

**Keywords:** Hormone receptors, Homeostasis, Diabetes

## Abstract

Glucagon has emerged as a key regulator of extracellular amino acid (AA) homeostasis. Insufficient glucagon signaling results in hyperaminoacidemia, which drives adaptive proliferation of glucagon-producing α cells. Aside from mammalian target of rapamycin complex 1 (mTORC1), the role of other AA sensors in α cell proliferation has not been described. Here, using both genders of mouse islets and glucagon receptor (*gcgr*)-deficient zebrafish (*Danio rerio*), we show α cell proliferation requires activation of the extracellular signal-regulated protein kinase (ERK1/2) by the AA-sensitive calcium sensing receptor (CaSR). Inactivation of CaSR dampened α cell proliferation, which was rescued by re-expression of CaSR or activation of Gq, but not Gi, signaling in α cells. CaSR was also unexpectedly necessary for mTORC1 activation in α cells. Furthermore, coactivation of Gq and mTORC1 induced α cell proliferation independent of hyperaminoacidemia. These results reveal another AA-sensitive mediator and identify pathways necessary and sufficient for hyperaminoacidemia-induced α cell proliferation.

## Abstract (web summary)

Insufficient glucagon signalling results in hyperaminoacidemia, which drives adaptive proliferation of glucagon-producing α cells. Here the authors report that the amino acid sensitive calcium sensing receptor (CaSR) is necessary for α cell proliferation via Gq signalling during hyperaminoacidemia.

## Introduction

Amino acids (AAs) are one of the main building blocks and fuel sources of life. As such, plasma AA levels are tightly regulated and remain constant during protein restriction and short-term fasting^1,2^. Glucagon has emerged as a key hormone regulating plasma AA homeostasis in recent years^3–5^. Glucagon stimulates AA catabolism by activating ureagenesis and gluconeogenesis in the liver^5^. *Gcgr*−/− mice have a two- to tenfold increase in individual AAs except for Trp and Phe and a more than 3-fold increase in the total plasma AA concentration (hyperaminoacidemia), which in turn induces proliferation of glucagon-producing α cells^6–9^. Liver-specific inactivation of *Gcgr*^10^ or its downstream gene *Gnas1*^11^ also leads to α cell hyperplasia in mice. Persistent loss of glucagon signaling eventually results in glucagonoma, tumors of α cells in mice and humans^12–14^. These recent studies thus reveal an α cell-liver axis in plasma AA homeostasis. Glucagon receptor antagonism (GRA) has been shown to be very effective in lowering blood glucose in preclinical diabetes models and to improve glycemic control when used as an adjunct to insulin therapy in T1D patients^15^. This axis is a roadblock for clinical use of GRA therapy for diabetes^16^. However, how hyperaminoacidemia induces α cell proliferation is not fully understood.

A few mediators have been implicated in hyperaminoacidemia-induced α cell proliferation. Unsurprisingly, the AA sensor mTORC1 is necessary for the hyperaminoacidemia-induced α cell proliferation^6,7,9^. The preferential response of α cells to hyperaminoacidemia is due in part to a mTORC1-dependent induction of the glutamine transporter *Slc38a5* as its ablation dampens the proliferation^6,7^. In *Slc38a5*−/− mice and in humans where SLC38A5 induction in α cells is absent, however, hyperaminoacidemia still causes α cell proliferation^6,7^. Furthermore, although genetic activation of mTORC1 alone in mouse α cells induces α cell proliferation in adult mice, no change is detected in newborns^17^. The increased α cell proliferation in adult mice with mTORC1 activation may be secondary to hepatic glucagon resistance caused by mTORC1-induced hypersecretion of glucagon. Therefore, other cell-intrinsic signaling pathways may also be necessary for compensatory α cell proliferation.

Some members of the class C G-protein-coupled receptor (GPCR) act as broad-spectrum amino acid sensors that activate intracellular signals^18^. In particular, CaSR is a class C GPCR that senses L-AA and Ca^2+^, among other endogenous ligands^19,20^. After expression cloning from a bovine parathyroid mRNA library^21^, and subsequent cloning by homology screening of a human parathyroid cDNA library^22^, loss of function and gain of function mutations of the CaSR respectively were subsequently found to cause familial hypocalciuric hypercalcemia and autosomal dominant hypocalcemia in human kindreds, respectively^23^. In addition to calcitropic tissues (for example, parathyroid glands, kidney, and breast), CaSR is also expressed in noncalcitropic tissues including enteroendocrine and pancreatic endocrine cells^19^. In the pancreatic islet, CaSR is highly expressed in α and β cells^24,25^ and regulates pancreatic endocrine cell secretion^25^. CaSR couples to multiple heterotrimeric G-protein subtypes (Gq/11, Gi/o, and G12/13) to activate intracellular signal transduction cascades^26^. These cascades lead to increased cytosolic calcium concentrations and activation of the mitogen-activated protein kinase (MAPK) pathway, which ultimately result in phosphorylation and activation of ERK1/2^26^. Notably, CaSR has also been shown to regulate cell proliferation^27^. Its mutation or aberrant activation is associated with various types of cancer and altered pancreatic islet mass^28,29^. However, whether CaSR contributes to hyperaminoacidemia-induced pancreatic α cell proliferation remains unclear.

In the present study, using zebrafish, a mouse α cell line, and primary mouse islets as models, we set out to determine the molecular mechanism of hyperaminoacidemia-induced pancreatic α cell proliferation. We deciphered that the CaSR-Gq-ERK1/2 pathway is another mediator of hyperaminoacidemia-induced pancreatic α cell proliferation. We show that CaSR-activated Mek1/2 also contributes to mTORC1 activation. Importantly, coactivation of CaSR-Gq and mTORC1 in α cells causes proliferation in the *gcgr*-intact zebrafish. These results identify the major pathways that are necessary and sufficient for hyperaminoacidemia-induced α cell proliferation and illustrate a previously unidentified physiological role of CaSR.

## Results

### Loss of GCGR causes hyperaminoacidemia and mTORC1 activation in zebrafish α cells

**Fig. 1:**
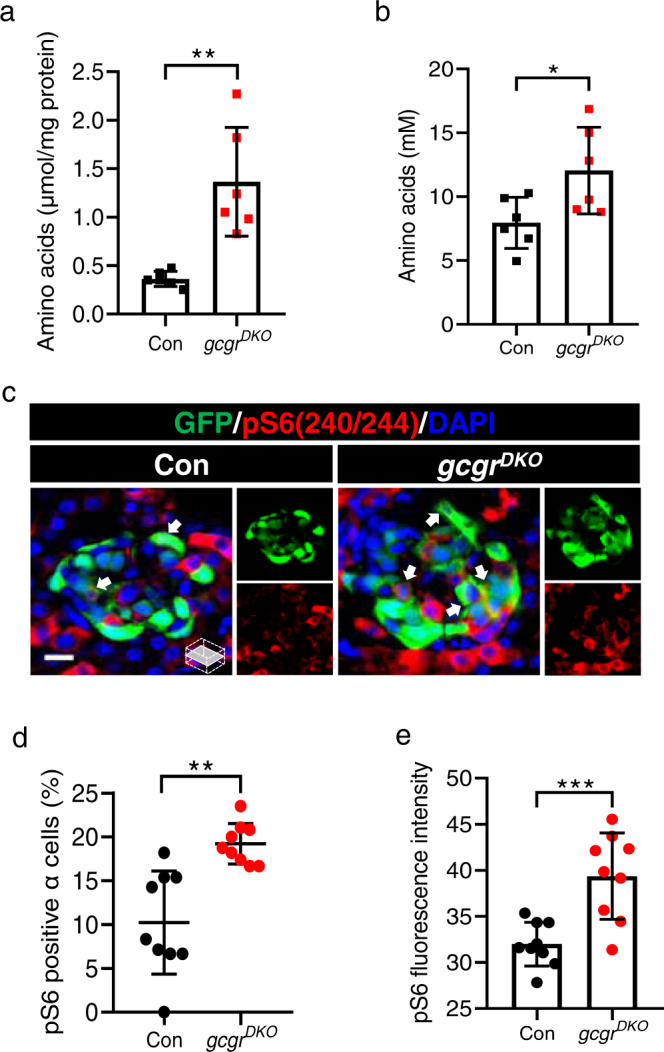
Elevated free amino acids and α cell mTORC1 activity in gcgrDKO zebrafish. **a** The free amino acid levels of control and *gcgr*^*DKO*^ larvae at 5 dpf, normalized by total protein concentration (Data represent means ± SD, *n* = 6, 40 fish/replicate, for each group). **b** Serum-free amino acid levels of control and *gcgr*^*DKO*^ zebrafish adults (data represent the means ± SD, *n* = 6 for each group). **c** Representative images of pS6(240/244) and GFP immunofluorescence in islet sections of control and *gcgr*^*DKO*^ larvae at 5 dpf. The pS6(240/244) signal in α cell is indicated by arrows, primary antibody: anti-pS6 (Ser240/244) (1:300, rabbit); secondary antibody: Alexa Fluor 568 (1:1000, goat anti-rabbit) (scale bar, 8 μm). **d** Quantification of the percentage of pS6(240/244) positive α cells in control and *gcgr*^*DKO*^ larvae at 5 dpf (data represent the means ± SD, *n* = 9 for each group). **e** Quantification of the pS6(240/244) fluorescence intensity in α cells of control and *gcgr*^*DKO*^ larvae at 5 dpf (data represent the means ± SD, *n* = 9 for each group). *P < 0.05, **P < 0.01, ***P < 0.001 (two-tailed unpaired t test, the quantifications represent individual islet sections). Source data are provided as a Source Data file.

We previously demonstrated that the α cell-liver axis is conserved in zebrafish. In zebrafish lacking both glucagon receptor a (*gcgra*) and glucagon receptor b (*gcgrb*) (*gcgr*^*DKO*^), there are more α cells due to increased proliferation^30^. In addition, the hyperplasia is rapamycin-sensitive and depends on *slc38a5b*^7^, similar to *Gcgr*−/− mice^6,7,16^. *Gcgr*−/− mice also have hyperaminoacidemia and increased mTORC1 in cells^6–9^. Whether this is true in *gcgr*^*DKO*^ zebrafish has not been determined. To fully characterize the phenotypic conservation, we determined the free AA levels in whole larvae and AA concentration in adult sera as well as the mTORC1 activation in α cells of *gcgr*^*DKO*^ zebrafish. Compared to the control groups, the *gcgr*^*DKO*^ zebrafish had increased free AA content in larvae and serum AA concentration in adults (Fig. 1a, b). The activity of mTORC1, as indicated by the extent and intensity of the immunofluorescence signal of serine 240/244 phosphorylation of S6 ribosomal protein (pS6), was also increased in the α cells of *gcgr*^*DKO*^ zebrafish (Fig. 1c–e). These data further confirm that the *gcgr*^*DKO*^ zebrafish is a valid model for investigating the mechanisms of hyperaminoacidemia-induced α cell proliferation.

### Activation of mTORC1 is insufficient for α cell proliferation in zebrafish

**Fig. 2:**
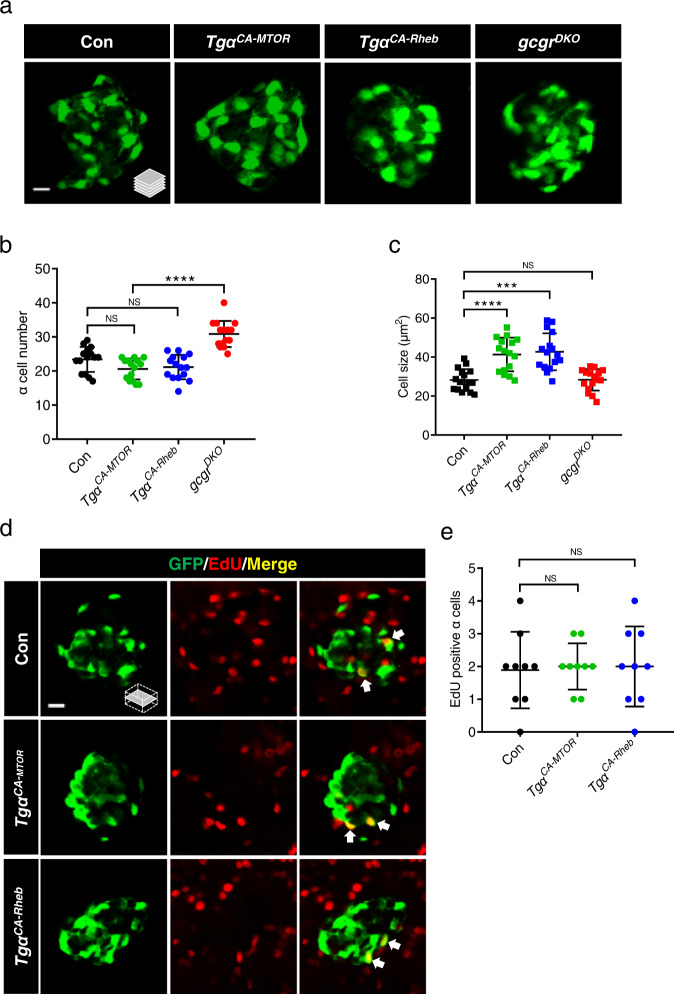
Activation of mTORC1 is insufficient for α cell proliferation. **a** Representative images of α cells in control, *Tgα*^*CA-MTOR*^ (*Tg(gcga:MTOR*^*L1460P*^*, cryaa:tagRFP)*), *Tgα*^*CA-Rheb*^ (*Tg(gcga:Rheb*^*S16H*^*, cryaa:tagYFP)*), and *gcgr*^*DKO*^ (*gcgra−/−;gcgra−/−*) larvae at 5 dpf (scale bar, 10 μm). **b** Quantification of α cell number in control, *Tgα*^*CA-MTOR*^, *Tgα*^*CA-Rheb*^ and *gcgr*^*DKO*^ larvae at 5 dpf (data represent the means ± SD, *n* = 15 for each group). **c** Quantification of α cell size in control, *Tgα*^*CA-MTOR*^, *Tgα*^*CA-Rheb*^ and *gcgr*^*DKO*^ larvae at 5 dpf (Data represent means ± SD, *n* = 16 for each group). **d** Representative images of EdU staining in control, *Tgα*^*CA-MTOR*^ and *Tgα*^*CA-Rheb*^ larvae at 5 dpf. EdU (red)-positive α cells are indicated by arrows (scale bar, 10 μm). **e** Quantification of EdU-positive α cell numbers in control, *Tgα*^*CA-MTOR*^ and *Tgα*^*CA-Rheb*^ larvae at 5 dpf (data represent the means ± SD, n = 9 for each group). ***P < 0.001, ****P < 0.0001, NS indicates no significant difference (one-way ANOVA, Tukey’s multiple comparisons test, the quantifications represent individual islet sections). Source data are provided as a Source Data file.

Whether mTORC1 activation is sufficient for α cell proliferation is unknown. To answer this question, we established two transgenic lines, *Tg(gcga:MTOR*^*L1460P*^*, cryaa:tagRFP)* and *Tg(gcga:Rheb*^*S16H*^*, cryaa:YFP)* (*Tgα*^*CA-MTOR*^ and *Tgα*^*CA-Rheb*^ hence forward), that express a constitutively active mTOR^L1460P^ (CA-MTOR) or Rheb^S16H^ (CA-Rheb), an upstream activator of mTORC1, only in α cells. Each transgenic construct also included a transgene that directs lens-specific expression of a fluorescent protein to facilitate carrier identification^31^. Throughout this study, we used the *Tg(gcga:GFP)* transgene to facilitate α cell counting as was done previously^30^. We assessed the total and EdU-positive α cell numbers in these transgenic fish. However, there was no significant change in the number of either total α cells or EdU-positive α cells in either of the transgenic lines at 5 days postfertilization (dpf) (Fig. 2a, b, d and e). Nonetheless, α cells in both transgenic lines exhibited hypertrophy compared to the control (Fig. 2a, c), confirming mTORC1 activation. This was further evidenced by an overt increase in the immunofluorescence signal of pS6 in the α cells of both transgenic lines (Supplementary Fig. 1a–c) independent of an increase in free AA content (Supplementary Fig. 1d). These results indicate that mTORC1 activation leads to α cell hypertrophy but not hyperplasia at 5 days postfertilization (dpf). However, adult *Tgα*^*CA-MTOR*^ fish had a significantly increased α cell area (Supplementary Fig. 1e, f) and also exhibited increased AA levels compared to the WT control (Supplementary Fig. 1g), likely a secondary effect of hyperglucagonemia and subsequent glucagon resistance. Since high concentrations of AA are sufficient to induce α cell proliferation in primary islets^6,7,9^, we hypothesize that other AA-sensitive pathway(s) may also be required for hyperaminoacidemia-induced α cell proliferation.

### CaSR is essential for hyperaminoacidemia-induced α cell proliferation in zebrafish and mouse islets

AAs are capable of stimulating certain GPCRs, which subsequently, can induce cell proliferation. There AA-sensitive GPCRs, *gprc6a*, *tas1r1*/*tas1r3,* and *casr* are expressed in mouse α cells^32^, respond to multiple L-AAs^33^, and have previously been implicated in the regulation of endocrine cell function^34–36^. We therefore first determined whether they are required for α cell hyperplasia in *gcgr*^*DKO*^ zebrafish and then validated the results in mouse α cell line and primary islets.

**Fig. 3:**
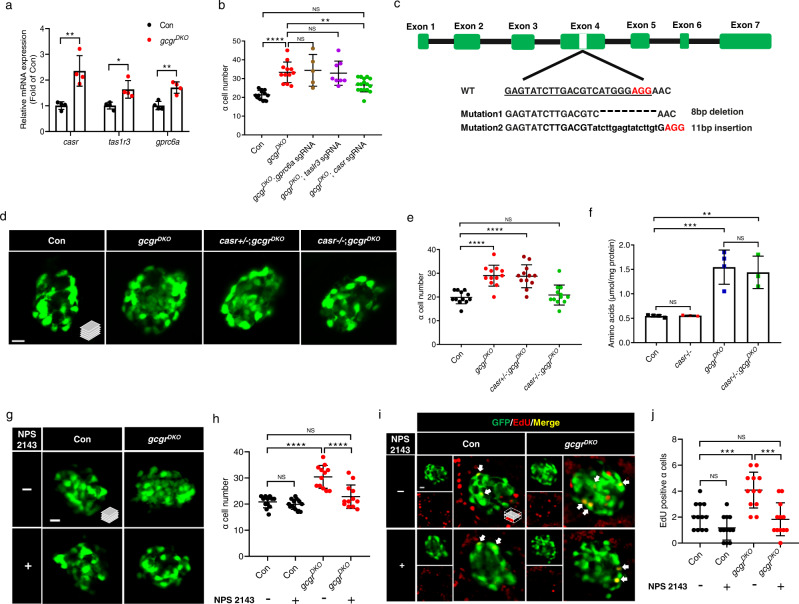
CaSR is required for α cell proliferation in *gcgr*^*DKO*^ zebrafish. **a** qPCR analysis of islet amino acid sensing GPCRs (*gprc6a*, *tas1r3*, *casr*) (data represent the means ± SD, *n* = 4 for each group). **b** Quantification of α cell number in 5 dpf larvae of control, *gcgr*^*DKO*^, and *gcgr*^*DKO*^ with knockdown of amino acid sensing GPCRs (*gprc6a, tas1r3, casr*) (Data represent means ± SD, *n* = 12, 12, 5, 8, 16 for each group). **c**. Diagram of *casr* CRISPR mutagenesis including target location, target sequence (underlined), PAM (red) and characterized mutations. Lower case letters indicate insertion. Hyphens represents deletion. **d**. Representative images of α cells in control, *gcgr*^*DKO*^, *cas*+/-;*gcgr*^*DKO*^ and *casr*−/−;*gcgr*^*DKO*^ larvae at 5 dpf (scale bar, 10 μm). **e** Quantification of α cell numbers in control, *gcgr*^*DKO*^, *cas*+/-;*gcgr*^*DKO*^ and *casr*−/−;*gcgr*^*DKO*^ larvae at 5 dpf (data represent the means ± SD, *n* = 12 for each group). **f** Free amino acid levels of control, *casr−/−*, *gcgr*^*DKO*^, and *casr*−/−;*gcgr*^*DKO*^ larvae at 5 dpf, normalized by total protein concentration (data represent the means ± SD, *n* = 3/4, 40 fish/replicate, for each group). **g** Representative images of α cells in control and *gcgr*^*DKO*^ larvae treated with 1 μM NPS 2143 or vehicle for 48 h at 5 dpf (scale bar, 10 μm). **h** Quantification of α cell numbers in control and *gcgr*^*DKO*^ larvae treated with 1 μM NPS 2143 or vehicle for 48 h at 5 dpf (data represent the means ± SD, *n* = 12 for each group). **i**. Representative images of EdU staining in control and *gcgr*^*DKO*^ larvae treated with 1 μM NPS 2143 or vehicle for 48 h at 5 dpf. EdU (red)-positive α cells are indicated by arrows (scale bar, 10 μm). **j**. Quantification of EdU positive α cell numbers in control and *gcgr*^*DKO*^ larvae treated with 1 μM NPS 2143 or vehicle for 48 h at 5 dpf (data represent the means ± SD, *n* = 12 for each group). *P < 0.05, **P < 0.01, ***P < 0.001, ****P < 0.0001, NS means no significant difference (two-tailed unpaired *t* test was used for Fig. 2a. One-way ANOVA and Tukey’s multiple comparisons test were used for Fig. 2b, e, f, h and j; the quantifications represent individual islet sections). Source data are provided as a Source Data file.

To determine whether the AA-sensitive GPCRs play a role in α cell proliferation in zebrafish, we first characterized the expression and observed that *casr*, *tas1r3,* and *gprc6a* were all upregulated in the islets of *gcgr*^*DKO*^ zebrafish, with *casr* increasing by a more than twofold at 5 dpf (Fig. 3a). *tas1r1* was not detectable in the islet at this stage. We then used efficient CRISPR mutagenesis to knock down *gprc6a*, *tas1r3*, and *casr* individually in *gcgr*^*DKO*^ zebrafish (Supplementary Fig. 2a-c). The Synthego ICE mutagenesis scores were 96, 83, and 95 for *gprc6a*, *tas1r3*, and *casr*, respectively (Supplementary Fig. 2a–c). The results revealed that only the *casr* knockdown significantly reduced the α cell number to that of the control group (Fig. 3b), suggesting that CaSR is involved in α cell proliferation. Hence, we established two independent *casr* mutant lines for cross-validation (Fig. 3c). Loss of *casr* function did not affect gross appearance or body length at 5 dpf (Supplementary Fig. 3a, b), the stage most of the analyses were performed in this study. As expected, the mutants had hypercalcemia at 7 dpf (Supplementary Fig. 3c) and impaired calcium deposition in the notochord at 10 dpf (Supplementary Fig. 3d, e). In 2-month-old adult zebrafish, *casr* mutants displayed skeletal dysplasia (Supplementary Fig. 3f, g) and marked growth retardation (Supplementary Fig. 3h, i). The mutant lines did not survive past 3 months of age. These results are reminiscent of *Casr*-deficient mice^37,38^, supporting that the mutations are loss of function.

We evaluated α cell number and proliferation in zebrafish *casr* mutants. Compared to wild-type and heterozygous (*casr*+*/-*) zebrafish, casr−/− zebrafish exhibited a significant decrease in the total number of α cells and EdU-labeled (proliferating or recently divided) α cells (Supplementary Fig. 4a-d). These results indicate that CaSR plays a role in α cell proliferation in normal aminoacidemia. To test whether CaSR is required for hyperaminoacidemia-induced α cell proliferation, we crossed the *casr* mutant into the *gcgr*^*DKO*^ background to generate the triple mutants (*casr*−/−;*gcgr*^*DKO*^). We found that the α cell number in 5-dpf *casr*−/−;*gcgr*^*DKO*^ fish was significantly reduced compared to that in *gcgr*^*DKO*^ fish and was similar to that in wild-type controls (Fig. 3d, e). The triple mutants were still hyperaminoacidemic (Fig. 3f). Furthermore, pharmacological inhibition of CaSR by 1 μM NPS 2143 also significantly suppressed α cell number and proliferation in *gcgr*^*DKO*^ zebrafish (Fig. 3g–j).

We used small molecule inhibitors on αTC1-6 cells and mouse islets to determine whether CaSR is also necessary for hyperaminoacidemia-induced α cell proliferation in mammalian α cells. As previously reported^6^, 4 mM glutamine was optimal for stimulating the proliferation of αTC1-6 cells (Supplementary Fig. 4e). To determine the Ca^2+^ sensitivity, we cultured the cells in 4 mM glutamine medium with different Ca^2+^ concentrations and found that the proliferation was Ca^2+^ sensitive (Supplementary Fig. 4f). Consistent with the essential role of CaSR, the proliferation was abolished by 2 µM NPS 2143 (Supplementary Fig. 4g). Previous studies have identified inorganic phosphate as a noncompetitive inhibitor of the CaSR^39,40^. We, therefore, tested whether proliferation is also phosphate sensitive by culturing αTC1-6 cells in the proliferation medium with 1.2 mM (physiological) or 5.6 mM phosphate. Unexpectedly, the higher phosphate concentration had no negative effect on high AA-stimulated proliferation (Supplementary Fig. 4h).

We then assessed the role of CaSR in high AA-stimulated α cell proliferation in primary mouse islets^7^. Using Calhex 231 to inhibit CaSR, we observed that HAA-induced α cell proliferation in primary mouse islets was significantly decreased (Supplementary Fig. 4i, j).

Together, these results indicate that CaSR is essential for hyperaminoacidemia-induced α cell proliferation in zebrafish, αTC1-6 cells, and mouse islets.

### Hyperaminoacidemia-induced α cell proliferation requires CaSR cell-autonomously

CaSR is broadly expressed. It is unknown whether the function of CaSR is α cell autonomous or non-α cell autonomous. We addressed this question both in zebrafish and mouse islets.

**Fig. 4:**
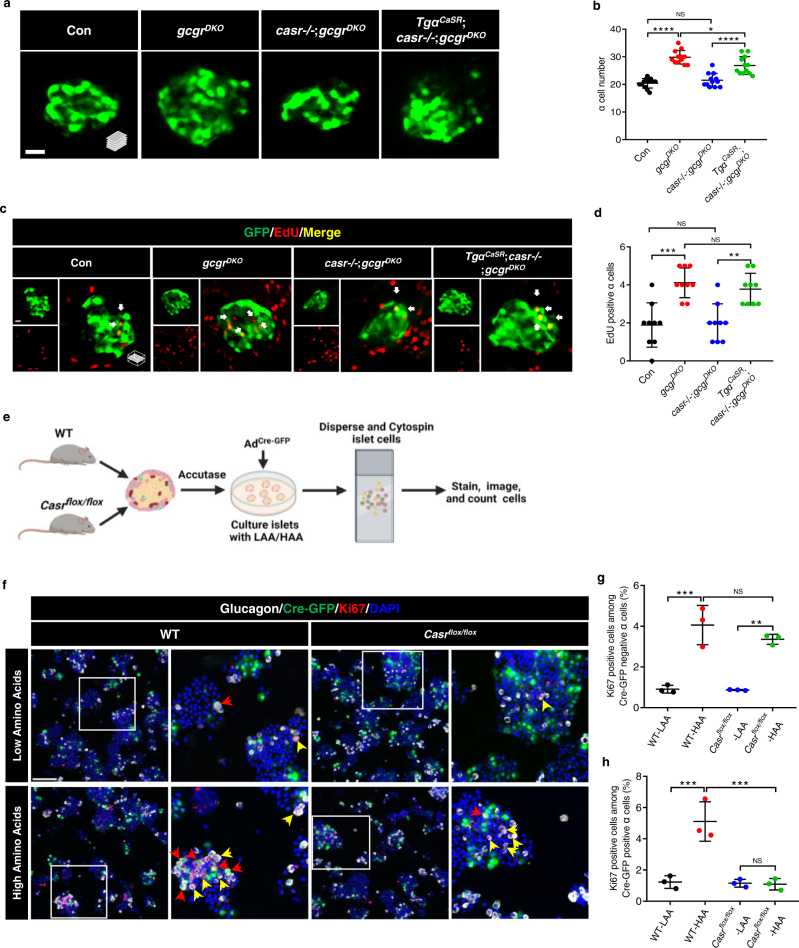
Hyperaminoacidemia-induced α cell proliferation requires CaSR cell-autonomously. **a** Representative images of α cells in control, *gcgr*^*DKO*^, *casr*−/−;*gcgr*^*DKO*^ and *Tgα*^*CaSR*^(*Tg(gcga:CASR, cryaa:tagRFP)*);*casr*−/−;*gcgr*^*DKO*^ larvae at 5 dpf (scale bar, 10 μm). **b** Quantification of α cell numbers in control, *gcgr*^*DKO*^, *casr*−/−;*gcgr*^*DKO*^ and *Tgα*^*CaSR*^;*casr*−/−;*gcgr*^*DKO*^ larvae at 5 dpf (data represent the means ± SD, *n* = 12 for each group). **c** Representative images of EdU staining in control, *gcgr*^*DKO*^, *casr*−/−;*gcgr*^*DKO*^ and *Tgα*^*CaSR*^;*casr*−/−;*gcgr*^*DKO*^ larvae at 5 dpf. EdU (Red) positive α cells are indicated by arrows (scale bar, 10 μm). **d** Quantification of EdU positive α cell number in control, *gcgr*^*DKO*^, *casr*−/−;*gcgr*^*DKO*^ and *Tgα*^*CaSR*^;*casr*−/−;*gcgr*^*DKO*^ larvae at 5 dpf (Data represent means ± SD, *n* = 9 for each group). **e** Schematic for *Casr* knockdown by Ad^Cre-GFP^ and α cell proliferation assays in the primary islets of *Casr*^*flox/flox*^ and WT mice (created with BioRender.com). **f** Representative images of cells from primary WT and *Casr*^*flox/flox*^ mouse islets cultured with LAA and HAA media after Ad^Cre-GFP^ transduction. Ki67 (red) and Cre-GFP (green) double-positive α cells are indicated by red arrows, Ki67 (red) single-positive α cells are indicated by yellow arrows, primary antibody: Anti-GFP (1:300, chicken)/anti-Glucagon (1:200, mouse)/anti-Ki67 (1:150, rabbit); secondary antibody: Alexa Fluor 488 (1:1000, goat anti-chicken)/Alexa Fluor 647 (1:1000, goat anti-mouse)/Alexa Fluor 568 (1:1,000, goat anti-rabbit) (scale bar, 150 μm). **g** Quantification of the percentage of Ki67 positive cells among the Cre-GFP negative α cells of WT and *Casr*^*flox/flox*^ mouse islets cultured with LAA and HAA media after Ad^Cre-GFP^ transduction (data represent the means ± SD, *n* = 3 for each group). **h** Quantification of the percentage of Ki67 positive cells among the Cre-GFP positive α cells of WT and *Casr*^*flox/flox*^ mouse islets cultured with LAA and HAA mediums after Ad^Cre-GFP^ transduction (Data represent means ± SD, *n* = 3 for each group). *P < 0.05, **P < 0.01, ***P < 0.001, ****P < 0.0001, NS indicates no significant difference (one-way ANOVA, Tukey’s multiple comparisons test, the quantifications represent individual islet sections). Source data are provided as a Source Data file.

We generated a transgenic zebrafish line, *Tg(gcga:CASR, cryaa:tagRFP)*, that re-expresses the human CASR gene (*CASR*)^41^ specifically in α cells. Compared to *casr*−/−;*gcgr*^*DKO*^ zebrafish, the total α cell number and the number of EdU-labeled α cells were restored in *Tg(gcga:CASR, cryaa:tagRFP)* (*Tgα*^*CaSR*^);*casr*−/−;*gcgr*^*DKO*^ zebrafish, to the level of the *gcgr*^*DKO*^ zebrafish (Fig. 4a–d).

We then transduced primary islets of wild-type and *Casr*^*flox/flox*^ mice^38^ with CRE-GFP-expressing adenoviruses (Ad^Cre-GFP^) and cultured the transduced islets in HAA/LAA media for 3 days to assess α cell proliferation (Fig. 4e). Approximately 40% of α cells were transduced in all conditions (Supplementary Fig. 4k), allowing for comparison of transduced (Cre-GFP positive) and nontransduced (Cre-GFP negative) cells within the same condition. Compared to LAA, HAA induced α cell proliferation in transduced and non-transduced α cells in islets from WT mice (Fig. 4f-h), indicating that the expression of Cre-GFP did not affect the proliferative response to HAA. In islets from *Casr*^*flox/flox*^ mice, the HAA-induced proliferation was unaffected in nontransduced α cells but was abolished in transduced α cells (Fig. 4f-h). As a result, the total HAA-induced α cell proliferation rates in virus-treated *Casr*^*flox/flox*^ islets were significantly decreased compared to those in WT islets (Supplementary Fig. 4l).

Together, these results suggest that CaSR functions cell-autonomously in hyperaminoacidemia-induced α cell proliferation and that this function is conserved in vertebrates.

### Gq mediates hyperaminoacidemia-induced α cell proliferation in zebrafish

**Fig. 5:**
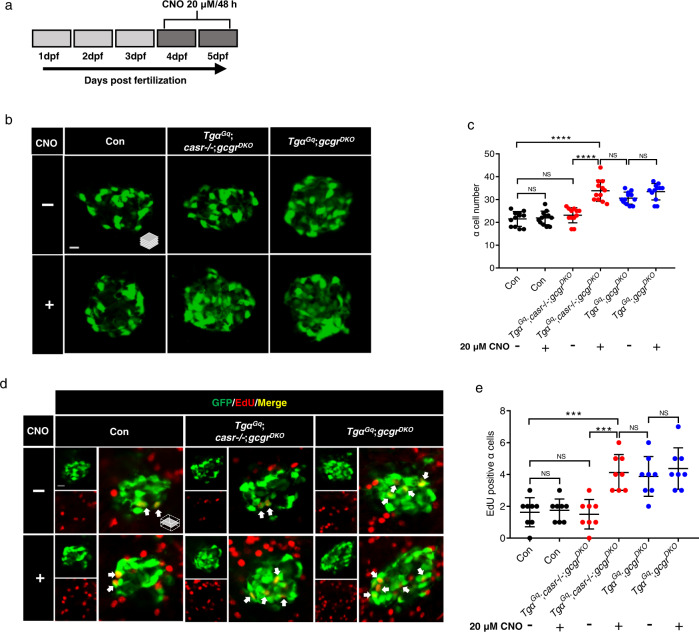
Activation of a Gq-DREADD in α cells restores proliferation in *casr−/−*;*gcgr*^*DKO*^ zebrafish. **a** Diagram of CNO treatment, 20 μM for 48 h. **b** Representative images of α cells in control, *Tgα*^*Gq*^(*Tg(gcga:hM3Dq, cryaa:tagRFP)*);*casr*−/−;*gcgr*^*DKO*^, and *Tgα*^*Gq*^;*gcgr*^*DKO*^ larvae with 20 μM CNO or vehicle at 5 dpf (scale bar, 10 μm). **c** Quantification of α cell numbers at 5 dpf in control, *Tgα*^*Gq*^;*casr*−/−;*gcgr*^*DKO*^, and *Tgα*^*Gq*^;*gcgr*^*DKO*^ larvae treated with 20 μM CNO or vehicle for 48 h (data represent the means ± SD, *n* = 12 for each group). **d** Representative images of EdU staining at 5 dpf in control, *Tgα*^*Gq*^;*casr*−/−;*gcgr*^*DKO*^ and Tgα^Gq^; *gcgr*^*DKO*^ larvae treated with 20 μM CNO or vehicle for 48 h. EdU (red)-positive α cells are indicated by arrows (scale bar, 10 μm). **e** Quantification of EdU-positive α cell numbers at 5 dpf in control, *Tgα*^*Gq*^;*casr*−/−;*gcgr*^*DKO*^, and *Tgα*^*Gq*^;*gcgr*^*DKO*^ larvae treated with 20 μM CNO or vehicle for 48 h (data represent the means ± SD, *n* = 8 for each group). ***P < 0.001, ****P < 0.0001, NS indicates no significant difference (One-way ANOVA, Tukey’s multiple comparisons test, the quantifications represent individual islet sections). Source data are provided as a Source Data file.

We used designer receptors exclusively activated by designer drugs (DREADDs) to determine the CaSR downstream signaling pathways involved in hyperaminoacidemia-induced α cell proliferation in zebrafish^42^. CaSR can couple to Gq and Gi to activate intracellular signals^26,43^. To determine whether Gq or Gi mediates α cell proliferation, we established 2 transgenic zebrafish lines, *Tg(gcga:hM3Dq, cryaa:tagRFP)*, and *Tg(gcga:hM4Di, cryaa:tagRFP)* (*Tgα*^*Gq*^ and *Tgα*^*Gi*^). These engineered receptors can be activated by CNO (Clozapine N-oxide) to initiate Gq and Gi signaling, respectively. Both DREADDs contained an HA-tag. Anti-HA immunofluorescence confirmed that they were both expressed specifically on the plasma membrane of the α cells (Supplementary Fig. 5a, b). We bred these transgenes into the *casr*−/−;*gcgr*^*DKO*^ and *gcgr*^*DKO*^ zebrafish and treated the progeny with 20 μM CNO for 48 h from 3 to 5 dpf (Fig. 5a). CNO treatment restored α cell number and proliferation in the *Tgα*^*Gq*^;*casr*−/−;*gcgr*^*DKO*^ group to those of the *gcgr*^*DKO*^ group, but did not increase α cell number and proliferation further in the *Tgα*^*Gq*^;*gcgr*^*DKO*^ group (Fig. 5b–e). However, CNO did not increase either α cell number or α cell proliferation in the *Tgα*^*Gi*^;*casr*−/−;*gcgr*^*DKO*^ group (Supplementary Fig. 5c–f). Overall, these results suggest that activation of Gq, not Gi, signaling leads to α cell proliferation and that CaSR may signal through Gq to induce α cell proliferation in zebrafish.

### Synergism of the Gq and mTORC1 pathways is sufficient to induce α cell proliferation in zebrafish

**Fig. 6:**
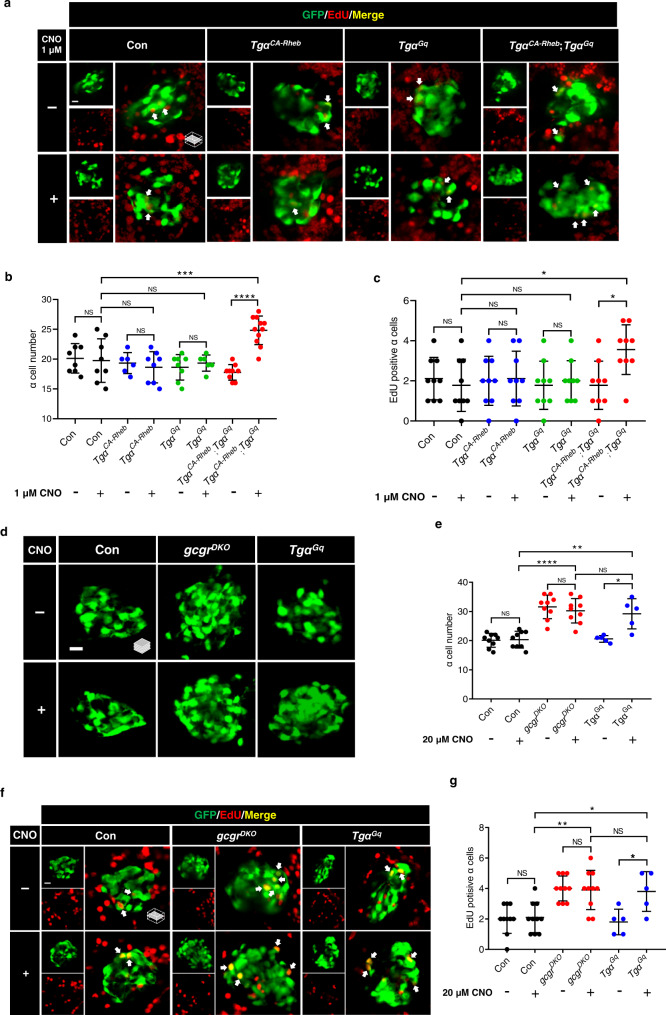
Synergism of the Gq and mTORC1 pathways is sufficient to induce α cell proliferation. **a** Representative images of EdU staining in control, *Tgα*^*CA-Rheb*^, *Tgα*^*Gq*^, and *Tgα*^*CA-Rheb*^;*Tgα*^*Gq*^ larvae treated with 1 μM CNO or vehicle for 48 h at 5 dpf. EdU (red)-positive α cells are indicated by arrows (scale bar, 10 μm). **b** Quantification of α cell numbers in control, *Tgα*^*CA-Rheb*^, *Tgα*^*Gq*^, and *Tgα*^*CA-Rheb*^;*Tgα*^*Gq*^ larvae treated with 1 μM CNO or vehicle for 48 h at 5 dpf. (data represent the means ± SD, *n* = 8, 8, 6, 8, 8, 6, 9, and 11 for each group, respectively). **c** Quantification of EdU-positive α cell numbers in control, *Tgα*^*CA-Rheb*^, *Tgα*^*Gq*^, and *Tgα*^*CA-Rheb*^;*Tgα*^*Gq*^ larvae treated with 1 μM CNO or vehicle for 48 h at 5 dpf (data represent the means ± SD, *n* = 9 for each group). **d** Representative images of α cells in control, *gcgr*^*DKO*^, and *Tgα*^*Gq*^ larvae treated with 20 μM CNO or vehicle for 48 h at 5 dpf (scale bar, 10 μm). **e** Quantification of α cell numbers in control, *gcgr*^*DKO*^ and *Tgα*^*Gq*^ larvae treated with 20 μM CNO or vehicle for 48 h at 5 dpf (data represent the means ± SD, *n* = 9, 9, 9, 9, 5, and 5 for each group, respectively). **f** Representative images of EdU staining in control, *gcgr*^*DKO*^ and Tgα^Gq^ larvae treated with 20 μM CNO or vehicle for 48 h at 5 dpf. EdU (red)-positive α cells are indicated by arrows (scale bar, 10 μm). **g** Quantification of EdU-positive α cell numbers in control, *gcgr*^*DKO*^ and *Tgα*^*Gq*^ larvae treated with 20 μM CNO or vehicle for 48 h at 5 dpf (data represent the means ± SD, *n* = 10, 10, 10, 10, 5, and 5 for each group, respectively). *P < 0.05, **P < 0.01, ***P < 0.001, ****P < 0.0001, NS indicates no significant difference (one-way ANOVA, Tukey’s multiple comparisons test, the quantifications represent individual islet sections). Source data are provided as a Source Data file.

To determine whether activation of the mTORC1 and Gq pathways is sufficient to cause α cell proliferation independent of hyperaminoacidemia, we crossed *Tgα*^*Gq*^ zebrafish with *Tgα*^*CA-Rheb*^ zebrafish. We treated the progeny with 1 µM or 20 µM CNO for 48 hours from 3 to 5 dpf to activate the Gq pathway in α cells. Activation of the Gq pathway at the lower CNO concentration, 1 µM, was insufficient to cause α cell proliferation in *Tgα*^*Gq*^ fish, but significantly increased the α cell number and proliferation level in *Tgα*^*Gq*^;*Tgα*^*CA-Rheb*^ fish without altering the free amino acid levels (Fig. 6a–c; Supplementary Fig. 6a). CNO (1 µM) treatment also increased the islet expression of glucagon a (*gcga*) and glucagon b (*gcgb*) in the *Tgα*^*Gq*^;*Tgα*^*CA-Rheb*^ fish (Supplementary Fig. 6b, c). These results suggest that the Gq and mTORC1 pathways act synergistically to induce α cell proliferation. Interestingly, at the higher concentration CNO, 20 µM, was sufficient to increase α cell number and proliferation in *gcgr*-intact *Tgα*^*Gq*^ fish to the same level as the *gcgr*^*DKO*^ group (Fig. 6d–g). This degree of activation is likely supraphysiological as the ligand concentration is over 1000-fold of its EC50 (5.4 ± 3.1 nM)^44^. This finding indicates that Gq hyperactivation can be sufficient for hyperaminoacidemia-independent α cell proliferation in zebrafish but that Gq and mTORC1 co-activation is likely to be required for the hyperaminoacidemia-dependent cell response under physiological conditions.

### CaSR-Gq induces α cell proliferation via the ERK1/2 pathway

**Fig. 7:**
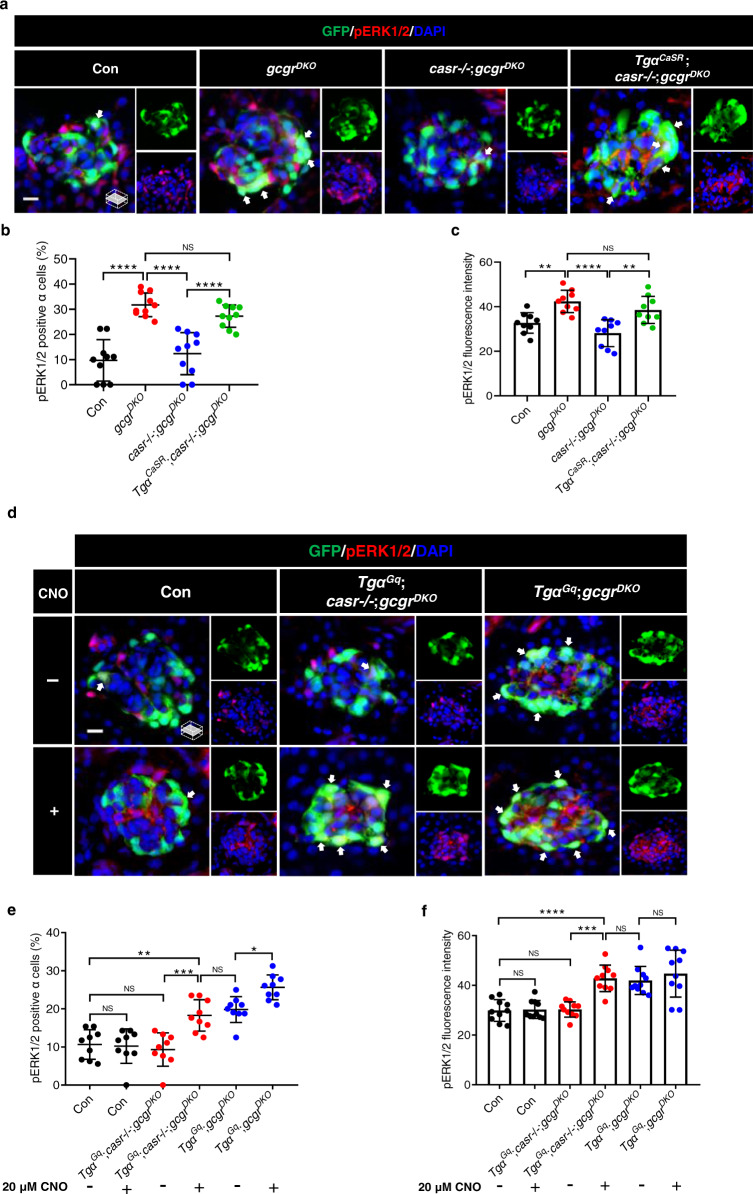
CaSR-Gq induces α cell proliferation via the ERK1/2 pathway. **a** Representative images of pERK1/2 and GFP immunofluorescence in islet sections of control, *gcgr*^*DKO*^, *casr*−/−;*gcgr*^*DKO*^ and *Tgα*^*CaSR*^;*casr*−/−;*gcgr*^*DKO*^ larvae at 5 dpf. The pERK1/2 signal in α cells is indicated by arrows, primary antibody: Anti-pERK1/2 (Thr202/Tyr204) (1:150, rabbit); secondary antibody: Alexa Fluor 568 (1:1,000, goat anti-rabbit) (scale bar, scale bar, 8 μm). **b** Quantification of the percentage of pERK1/2 positive α cells in control, *gcgr*^*DKO*^, *casr*−/−;*gcgr*^*DKO*^ and *Tgα*^*CaSR*^;*casr*−/−;*gcgr*^*DKO*^ larvae at 5 dpf (data represent the means ± SD, *n* = 10 for each group). **c** Quantification of pERK1/2 fluorescence intensity in α cell of control, *gcgr*^*DKO*^, *casr*−/−;*gcgr*^*DKO*^, and *Tgα*^*CaSR*^;*casr*−/−;*gcgr*^*DKO*^ larvae at 5 dpf (Data represent means ± SD, *n* = 9 for each group). **d** Representative images of pERK1/2 and GFP immunofluorescence in islet sections of control, *Tgα*^*Gq*^;*casr*−/−;*gcgr*^*DKO*^ and *Tgα*^*Gq*^;*gcgr*^*DKO*^ larvae at 5 dpf. The larvae were treated with 20 μM CNO or vehicle for 48 h. The pERK1/2 signal in α cells is indicated by arrows, primary antibody: Anti-pERK1/2 (Thr202/Tyr204) (1:150, rabbit); secondary antibody: Alexa Fluor 568 (1:1000, goat anti-rabbit) (scale bar, 8 μm). **e** Quantification of the percentage of pERK1/2 positive α cells in control, *Tgα*^*Gq*^;*casr*−/−;*gcgr*^*DKO*^ and *Tgα*^*Gq*^;*gcgr*^*DKO*^ larvae treated with 20 μM CNO or vehicle for 48 h at 5 dpf (data represent the means ± SD, *n* = 9 for each group). **f** Quantification of the pERK1/2 fluorescence intensity in α cells of control, *Tgα*^*Gq*^;*casr*−/−;*gcgr*^*DKO*^ and *Tgα*^*Gq*^;*gcgr*^*DKO*^ larvae treated with 20 μM CNO or vehicle for 48 h at 5 dpf (data represent the means ± SD, *n* = 10 for each group). *P < 0.05, **P < 0.01, ***P < 0.001, ****P < 0.0001, NS indicates no significant difference (one-way ANOVA, Tukey’s multiple comparisons test, the quantifications represent individual islet sections). Source data are provided as a Source Data file.

GPCR coupling of Gq activates multiple intracellular transduction cascades^26^. Pertinent to cell proliferation is the MAPK pathway, which leads to phosphorylation and consequent nuclear translocation of ERK1/2^45^. Hence, we next explored whether ERK1/2 is activated in α cells by hyperaminoacidemia in zebrafish. We found that the number of pERK1/2 positive α cells and nuclear pERK1/2 signal intensity were markedly increased in the *gcgr*^*DKO*^ group compared to that of the control group (Fig. 7a–c). The increase in pERK1/2 positive α cells and nuclear pERK1/2 signal intensity depended on *casr* as they were decreased 62.5 ± 7.9% and 32.9 ± 5.4% in the *casr*−/−;*gcgr*^*DKO*^ group, respectively (Fig. 7a–c). Re-expression of CaSR restored the increase in pERK1/2 positive α cells and nuclear pERK1/2 signal intensity in *Tgα*^*CaSR*^;*casr*−/−;*gcgr*^*DKO*^ zebrafish (Fig. 7a–c). These results indicate that hyperaminoacidemia-induced activation of CaSR activates ERK1/2. To determine whether CaSR activates ERK1/2 through Gq, we assayed the effect of Gq activation on pERK1/2 levels in α cells by using DREADD. We found that 20 µM CNO treatment significantly increased the pERK1/2 positive α cell percentage and nuclear pERK1/2 signal intensity of *Tgα*^*Gq*^;*casr*−/−;*gcgr*^*DKO*^ fish to the same levels as the *gcgr*^*DKO*^ fish (Fig. 7d–f). Interestingly, CNO even further increased the pERK1/2 positive α cell percentage further in the *Tgα*^*Gq*^;*gcgr*^*DKO*^ group (Fig. 7d, e). However, the Gi-DREADD only increased the percentage of pERK1/2 positive α cells, while the nuclear pERK1/2 translocation was impaired as the pERK1/2 signal intensity was unchanged in *Tgα*^*Gi*^;*casr*−/−;*gcgr*^*DKO*^ fish treated with CNO (Supplementary Fig. 7a–c). These results indicate that Gq, but not Gi, activation in α cells was sufficient to increase nuclear ERK1/2, which is critical for cell proliferation. Therefore, hyperaminoacidemia activates the CaSR-Gq-ERK1/2 pathway in the α cells of zebrafish.

**Fig. 8:**
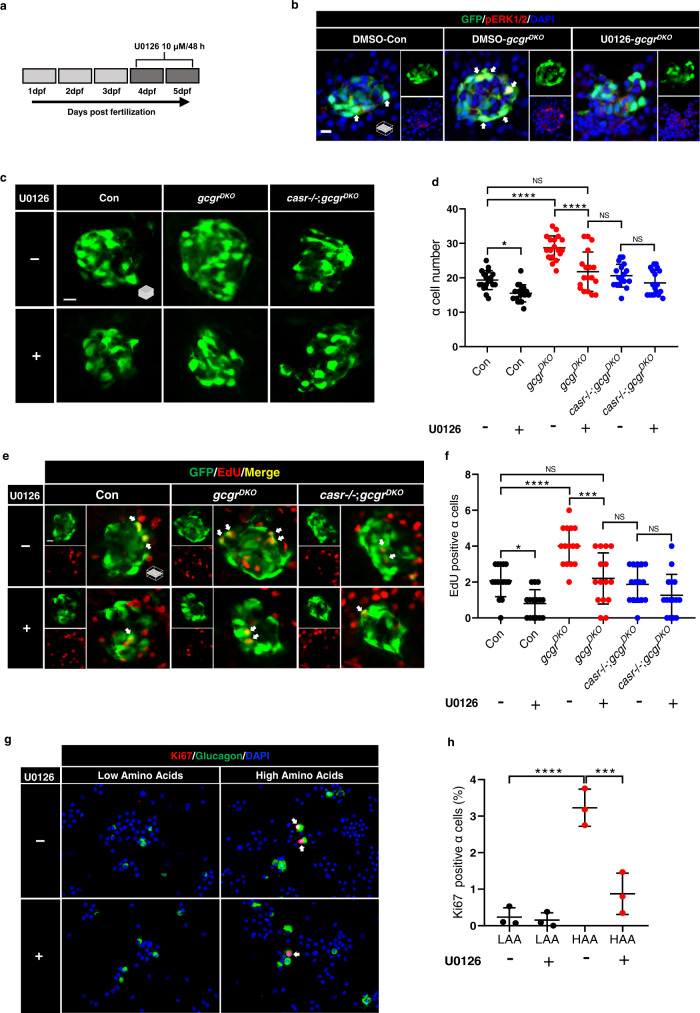
Mek1/2 activation is necessary for α cell proliferation. **a** Diagram of 48-h treatment with 10 μM U0126. **b**. Representative images of pERK1/2 and GFP immunofluorescence in islet sections of control and *gcgr*^*DKO*^ larvae treated with vehicle, 10 μM U0126 for 48 h at 5 dpf. The pERK1/2 signal in α cells is indicated by arrows, primary antibody: anti-pERK1/2 (Thr202/Tyr204) (1:150, rabbit); secondary antibody: Alexa Fluor 568 (1:1000, goat anti-rabbit) (scale bar, 7 μm). **c** Representative images of α cells at 5 dpf in control, *gcgr*^*DKO*^ and *casr*−/−;*gcgr*^*DKO*^ larvae treated with 10 μM U0126 or vehicle for 48 h (scale bar, 10 μm). **d** Quantification of α cell number at 5 dpf in control, *gcgr*^*DKO*^ and *casr*−/−;*gcgr*^*DKO*^ larvae treated with 10 μM U0126 or vehicle for 48 h (data represent the means ± SD, *n* = 18 for each group). **e** Representative images of EdU staining at 5 dpf in control, *gcgr*^*DKO*^ and *casr*−/−;*gcgr*^*DKO*^ larvae treated with 10 μM U0126 or vehicle for 48 h. EdU (Red) positive α cells are indicated by arrows (scale bar, 10 μm). **f** Quantification of EdU positive α cell numbers at 5 dpf in control, *gcgr*^*DKO*^ and *casr*−/−;*gcgr*^*DKO*^ larvae treated with 10 μM U0126 or vehicle for 48 h (data represent the means ± SD, *n* = 15 for each group). **g** Representative images of cells from primary mouse islets cultured in LAA and HAA medium with 10 μM U0126 or vehicle. Ki67 (red)-positive α cells are indicated by arrows, primary antibody: anti-Glucagon (1:200, mouse)/anti-Ki67 (1:150, rabbit); secondary antibody: Alexa Fluor 488 (1:1000, goat anti-mouse)/Alexa Fluor 568 (1:1000, goat anti-rabbit) (scale bar, 50 μm). **h** Quantification of the percentage of Ki67 positive α cells in dispersed cells from cultured islet cells in LAA and HAA media with 10 μM U0126 or vehicle. (data represent the means ± SD, *n* = 3 for each group). ***P < 0.001, ****P < 0.0001, NS indicates no significant difference (one-way ANOVA, Tukey’s multiple comparisons test, the quantifications represent individual islet sections). Source data are provided as a Source Data file.

It is unknown whether ERK1/2 activation is essential in hyperaminoacidemia-induced α cell proliferation. To answer this question, we utilized the Mek1/2 inhibitor, U0126^46^, to suppress ERK1/2 phosphorylation in control, *gcgr*^*DKO*^ and *casr*−/−;*gcgr*^*DKO*^ zebrafish for 48 h (10 μM dissolved in the egg water) (Fig. 8a). ERK1/2 is the only known physiological substrate of Mek1/2^47^. As expected, U0126 decreased the nuclear pERK1/2 levels in α cells of *gcgr*^*DKO*^ islets (Fig. 8b). U0126 also reduced the α cell number and proliferation in *gcgr*^*DKO*^ fish to those of the control and *casr*−/−;*gcgr*^*DKO*^ groups (Fig. 8c–f). Notably, U0126 even reduced α cell number and proliferation in the control fish (Fig. 8c–f).

Furthermore, it is unknown whether Mek1/2 is also necessary for amino acid-induced α cell proliferation in mammals. Therefore, we examined the effect of U0126 on HAA-stimulated α cell proliferation in primary mouse islets^7^. We found that U0126 significantly decreased the percentage of Ki67 positive α cells in the mouse islets cultured in HAA medium (Fig. 8g, h). These results indicate that ERK1/2 activation is essential for hyperaminoacidemia-induced α cell proliferation.

Taken together, these findings suggest that CaSR-Gq mediates hyperaminoacidemia-induced α cell proliferation via Mek1/2-ERK1/2 in zebrafish and mouse islets.

### Hyperaminoacidemia-induced mTORC1 activation in α cells requires CaSR and Mek1/2 in zebrafish and mouse islets

Previous studies demonstrated an essential role of mTORC1 in hyperaminoacidemia-induced α cell proliferation^6–9^. Since α cell proliferation also depended on CaSR, we tested whether mTORC1 activation requires CaSR function in zebrafish and mice.

**Fig. 9:**
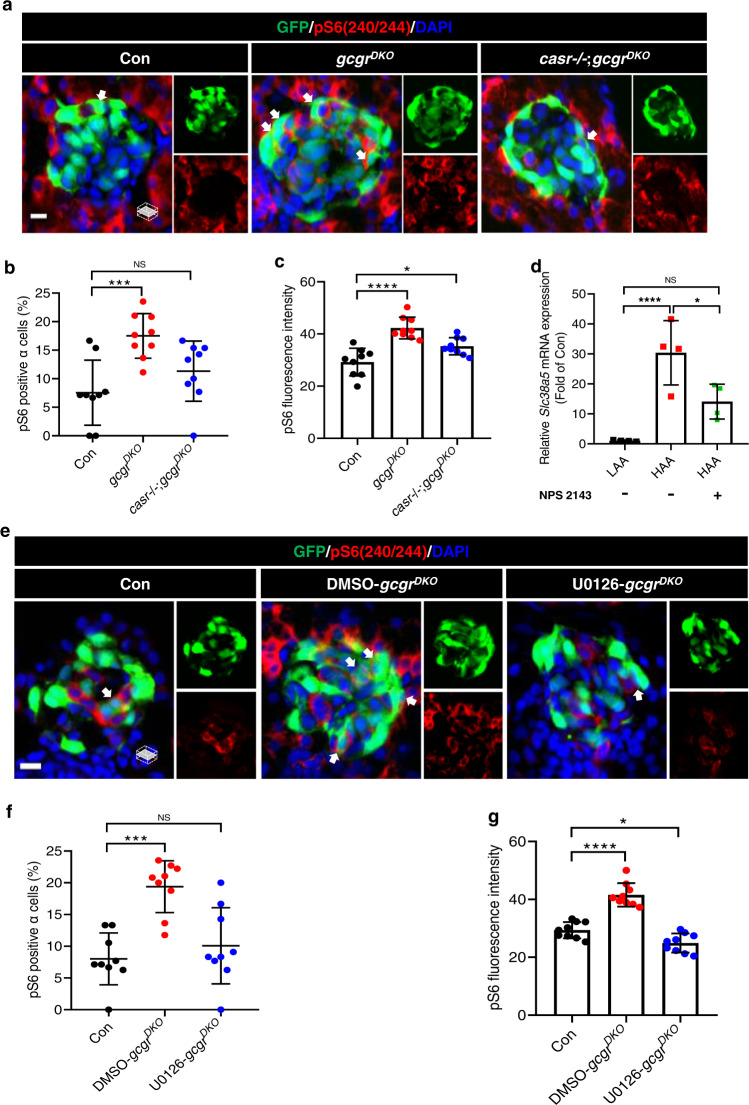
Hyperaminoacidemia-induced mTORC1 activation in α cells requires CaSR and Mek1/2. **a** Representative images of pS6(240/244) and GFP immunofluorescence in islet sections of control, *gcgr*^*DKO*^ and *casr*−/−;*gcgr*^*DKO*^ larvae at 5 dpf. The pS6(240/244) signal in α cells is indicated by arrows, primary antibody: Anti-pS6 (Ser240/244) (1:300, rabbit); secondary antibody: Alexa Fluor 568 (1:1000, goat anti-rabbit) (scale bar, 7 μm). **b** Quantification of the percentage of pS6(240/244) positive α cells in control, *gcgr*^*DKO*^, and *casr*−/−;*gcgr*^*DKO*^ larvae at 5 dpf (data represent the means ± SD, *n* = 9 for each group). **c** Quantification of the pS6(240/244) fluorescence intensity in α cells of control, *gcgr*^*DKO*^ and *casr*−/−;*gcgr*^*DKO*^ larvae at 5 dpf (data represent the means ± SD, *n* = 9 for each group). **d** qPCR analysis of *Slc38a5* in mouse islets cultured in LAA and HAA media with or without 0.5 µM NPS 2143 for 4 days. (data represent the means ± SD, *n* = 4 for each group). **e** Representative images of pS6(240/244) and GFP immunofluorescence in islet sections of control and *gcgr*^*DKO*^ larvae treated with 10 μM U0126 or vehicle for 48 h at 5 dpf. The pS6(240/244) signal in α cells is indicated by arrows, primary antibody: Anti-pS6 (Ser240/244) (1:300, rabbit); secondary antibody: Alexa Fluor 568 (1:1000, goat anti-rabbit) (scale bar, 7 μm). **f** Quantification of the percentage of pS6(240/244) positive α cells in control and *gcgr*^*DKO*^ larvae treated with 10 μM U0126 or vehicle for 48 h at 5 dpf (data represent the means ± SD, *n* = 9 for each group). **g** Quantification of the pS6(240/244) fluorescence intensity in α cells of control and *gcgr*^*DKO*^ larvae treated with 10 μM U0126 or vehicle for 48 h at 5 dpf (data represent the means ± SD, *n* = 9 for each group). *P < 0.05, ***P < 0.001, ****P < 0.0001, NS indicates no significant difference (one-way ANOVA, Tukey’s multiple comparisons test, the quantifications represent individual islet sections). Source data are provided as a Source Data file.

We first assessed mTORC1 activation in *casr*−/−;*gcgr*^*DKO*^ zebrafish. Unexpectedly, pS6 levels significantly decreased in *casr*−/−;*gcgr*^*DKO*^ fish when compared to *gcgr*^*DKO*^ fish (Fig. 9a–c), suggesting that CaSR is required for mTORC1 activation. To establish whether mTORC1 activation requires Mek1/2, we treated *gcgr*^*DKO*^ fish with U0126 (10 μM). U0126 decreased the percentage of pS6 positive α cells and the intensity of the α cell pS6 signal in *gcgr*^*DKO*^ fish to the same levels as in wild-type fish (Fig. 9e–g).

A downstream effect of mTORC1 activation in mice is the induction of *Slc38a*5^6,7^. We found that a 4-day culture of primary mouse islets in HAA, but not LAA, induced the expression of *Slc38a5* transcripts, which was dampened by 0.5 μM NPS 2143 (Fig. 9d).

These results suggest that hyperaminoacidemia-induced mTORC1 activation requires CaSR. At least in zebrafish, mTORC1 activation in α cells also requires the CaSR downstream effector Mek1/2.

### Gq hyperactivation-induced α cell proliferation requires mTORC1 activity in zebrafish

**Fig. 10:**
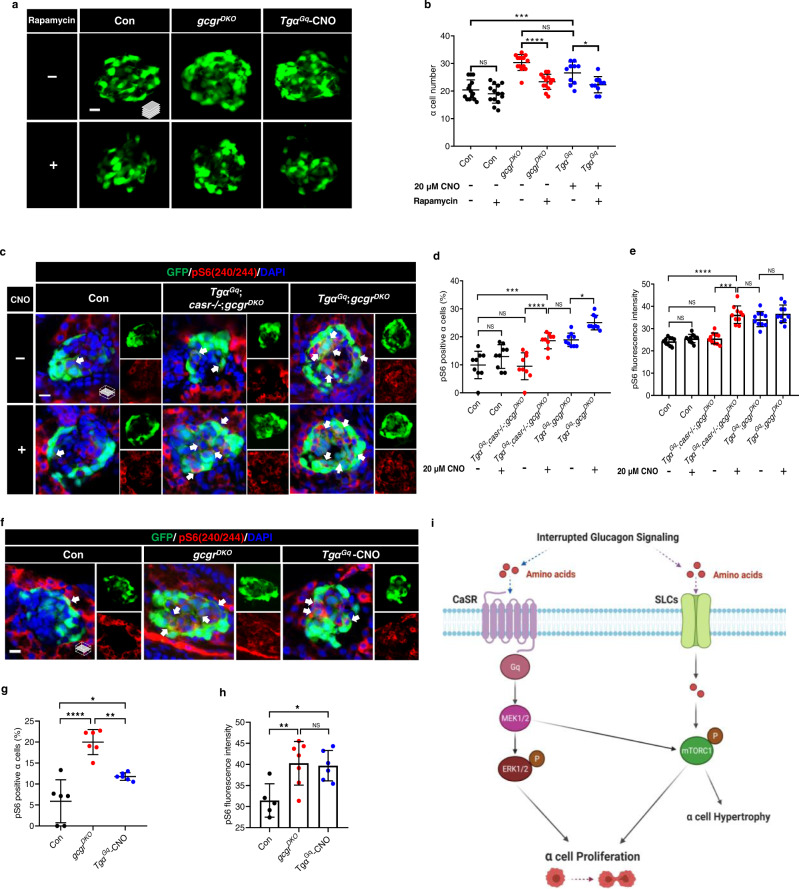
Gq hyperactivation-induced α cell proliferation requires mTORC1. **a** Representative images of α cells at 5 dpf in control, *gcgr*^*DKO*^ and *Tgα*^*Gq*^-CNO larvae treated with 2.5 μM rapamycin or vehicle for 48 h (scale bar, 10 μm). **b** Quantification of α cell number at 5 dpf in control, *gcgr*^*DKO*^ and *Tgα*^*Gq*^-CNO larvae treated with o 2.5 μM rapamycin or vehicle (data represent the means ± SD, *n* = 14, 14, 14, 14, 10, and 10 for each group, respectively). **c** Representative images of pS6(240/244) and GFP immunofluorescence in islet sections of control, *Tgα*^*Gq*^;*casr*−/−;*gcgr*^*DKO*^ and *Tgα*^*Gq*^;*gcgr*^*DKO*^ at 5 dpf. The larvae were treated with 20 μM CNO or vehicle. The pS6(240/244) signal in α cell is indicated by arrows, primary antibody: Anti-pS6 (Ser240/244) (1:300, rabbit); secondary antibody: Alexa Fluor 568 (1:1000, goat anti-rabbit) (scale bar, 7 μm). **d** Quantification of pS6(240/244) positive α cell percentages in control, *Tgα*^*Gq*^;*casr*−/−;*gcgr*^*DKO*^ and *Tgα*^*Gq*^;*gcgr*^*DKO*^ larvae treated with 20 μM CNO or vehicle at 5 dpf (Data represent means ± SD, *n* = 9 for each group). **e** Quantification of pS6(240/244) fluorescence intensity in α cells of control, *Tgα*^*Gq*^;*casr*−/−;*gcgr*^*DKO*^ and *Tgα*^*Gq*^;*gcgr*^*DKO*^ larvae with 20 μM CNO or vehicle at 5 dpf (data represent the means ± SD, *n* = 10 for each group). **f** Representative images of pS6(240/244) and GFP immunofluorescence in islet sections of control, *gcgr*^*DKO*^ and *Tgα*^*Gq*^ with 20 μM CNO at 5 dpf. The pS6(240/244) signal in α cells is indicated by arrows, primary antibody: anti-pS6 (Ser240/244) (1:300, rabbit); secondary antibody: Alexa Fluor 568 (1:1000, goat anti-rabbit) (scale bar, 7 μm). **g** Quantification of pS6(240/244) positive α cell percentages in control, *gcgr*^*DKO*^, and *Tgα*^*Gq*^ larvae treated with 20 μM CNO larvae at 5 dpf (data represent the means ± SD, *n* = 6 for each group). **h** Quantification of pS6(240/244) fluorescence intensity in α cells of control, *gcgr*^*DKO*^, and *Tgα*^*Gq*^ with 20 μM CNO larvae at 5 dpf (data represent the means ± SD, *n* = 5, 7, and 6 for each group, respectively). **i** Working model depicting the necessary and sufficient signaling pathways for hyperaminoacidemia-induced α cell proliferation: Interrupting glucagon signaling results in hyperaminoacidemia. The amino acid surge activates the CaSR-Gq-Mek1/2-ERK1/2 pathway. Concurrently, amino acids are transported into the cytoplasm and possibly lysosomes to activate mTORC1. mTORC1 is further activated by the CaSR-Gq-Mek1/2 pathway through an as-yet-unknown mechanism. The synergism between the CaSR-Gq and mTORC1 pathways mediates hyperaminoacidemia-induced α cell proliferation. *P < 0.05, **P < 0.01, ***P < 0.001, ****P < 0.0001, NS indicates no significant difference (one-way ANOVA and Tukey’s multiple comparisons test, the quantifications represent individual islet sections). Source data are provided as a Source Data file.

As Gq hyperactivation caused α cell hyperplasia in *gcgr*-intact zebrafish, we examined whether it also requires mTORC1. Indeed, rapamycin (2.5 µM) abolished α cell hyperplasia induced by Gq hyperactivation (induced by 20 μM CNO) in zebrafish (Fig. 10a, b). Consequently, Gq hyperactivation also increased pS6 levels in *Tgα*^*Gq*^;*casr*−/−;*gcgr*^*DKO*^ fish (Fig. 10c–e), and further amplified the percentage of pS6 positive α cells in *Tgα*^*Gq*^;*gcgr*^*DKO*^ fish compared to untreated controls (Fig. 10c, d). However, the Gi hyperactivation did not increase either the percentage of pS6 positive α cells or the intensity of the nuclear pS6 signal in *Tgα*^*Gi*^;*casr*−/−;*gcgr*^*DKO*^ fish (Supplementary Fig. 8a-c). Furthermore, the Gq hyperactivation (induced by 20 μM CNO) even increased pS6 levels in *gcgr*-intact zebrafish, although the percentage of pS6 positive α cells was still lower than that in the *gcgr*^*DKO*^ group (Fig. 10f–h). Therefore, Gq hyperactivation simultaneously activates ERK1/2 (Figs. 7 and 8) and mTORC1 (Figs. 9 and 10), and both are necessary for α cell proliferation in zebrafish.

Together, these data revealed that coactivation of Gq-ERK1/2 and mTORC1 is necessary and sufficient for inducing α cell proliferation. This study also uncovered a previously unidentified role of CaSR.

## Discussion

Glucagon is a key hormone that regulates plasma amino acid homeostasis by stimulating amino acid catabolism in the liver^3–5^. Impaired glucagon signaling leads to hyperglucagonemia and hyperaminoacidemia, which results in compensatory α cell proliferation^6–8,30,48^. Here, by combining gain-of-function and loss-of-function interventions, both in zebrafish and primary mouse islets, we found that the CaSR-Gq-ERK1/2 pathway is also essential for mediating hyperaminoacidemia-induced α cell proliferation. We showed that the synergy between the CaSR-Gq and mTORC1 pathways is sufficient to cause α cell proliferation independent of hyperaminoacidemia. We also demonstrated that mTORC1 activation is CaSR-Gq-Mek1/2 dependent during α cell proliferation. Therefore, we deciphered the major pathways that are necessary and sufficient for α cell proliferation and uncovered a previously unidentified physiological role for CaSR (Fig. 10i).

We have illustrated that activation of mTORC1 is insufficient to induce α cell proliferation in WT larvae. We observed that mTORC1-activated α cells are not hyperplastic but hypertrophic, similar to a recent study in which mTORC1 was activated by knocking out its constitutive inhibitor *Tsc2* in mouse α cells^17^. In that study, no α cell hyperplasia was observed in neonatal islets^17^, consistent with our results. However, adult *Tgα*^*CA-MTOR*^ zebrafish had significantly increased α cell area, similar to adult mice with α cell-specific mTORC1 activation^17^. In mice, this is due to the secondary effect of liver glucagon resistance resulting from increased glucagon secretion and hyperglucagonemia and is thus not an α cell-autonomous effect. The increased α cell area in adult *Tgα*^*CA-MTOR*^ fish is likely due to the same secondary effect as evidenced by hyperaminoacidemia (Supplementary Fig. 1e-g). We speculate that glucagon resistance has not been established in the liver of 5-dpf zebrafish. Of note, in the liver glucagon-resistant adult αTSC2^KO^ mice, blood amino acid levels were not significantly increased^17^. This contradicts our results and multiple published studies showing that hyperaminoacidemia is associated with liver glucagon resistance both in mice and humans^4,49^ and is a proposed biomarker for the condition. This discrepancy may result from differences in models and assays and from the partial glucagon resistance in the αTSC2^KO^ model.

We discovered that CaSR is α cell-autonomously required for hyperaminoacidemia-induced α cell proliferation. Since mTORC1 activation is insufficient to cause α cell proliferation cell-autonomously, we speculated a role of other amino acid-sensitive pathways and identified CaSR. Through loss of function and rescue studies, we demonstrated that CaSR is required cell-autonomously for hyperaminoacidemia-induced α cell proliferation. We further confirmed the necessary role of CaSR in high AA-induced α cell proliferation in primary mouse islets. Although it has been shown that CaSR is highly expressed in pancreatic endocrine cells^19,24,25^, we established a role for CaSR in hyperaminoacidemia-induced α cell proliferation for the first time. Previous studies also revealed a role for CaSR in other proliferating cell types in vitro^50,51^, while our study showed a necessary role for CaSR in α cell proliferation in vivo. Our results demonstrated functional conservation of CaSR, as human CaSR can rescue α cell proliferation in *casr*−/− zebrafish (Fig. 4c, d). This is not surprising given their 66% sequence identity.

Our study demonstrates an AA-sensitive function of CaSR in vivo under hyperaminoacidemia conditions. CaSR is known to be sensitive to AAs. Following the discovery of CaSR AA sensitivity in transfected cells^52^, the agonism of AAs on CaSR has been confirmed in native cells in vitro^53,54^. Importantly, CaSR deletion abolishes AA-induced secretion from enteroendocrine cells^55^. In addition, CaSR crystal structures reveal that AA binding to the orthosteric site in the Venus Flytrap domain causes a conformational change necessary for activating the receptor^56^. Unlike the frequent meal-associated changes in GI lumen AA concentration that the enteroendocrine cells experience, other CaSR-expressing endocrine cells in the body normally do not experience significant AA changes since blood amino acid levels are tightly maintained within a small range. Emerging evidence suggests a prominent role of α cells in maintaining AA homeostasis^4,5^. CaSR has not been reported to play a direct role in maintaining AA homeostasis.

L-Gln and L-Ala are important for hyperaminoacidemia-induced α cell proliferation^6,7^. This seems to disagree with the known CaSR pharmacological profiles of L-AAs^52^. L-Phe and L-Trp are the most potent^52,54^. However, the blood concentrations of L-Phe and L-Trp are less than 20% of the EC_50_ (2.2 mM) and are not increased by interrupting glucagon signaling^6,7,54^. In contrast, blood Gln and Ala concentrations are more than five times higher normally than L-Phe and L-Trp and are increased by more than fivefold by interrupting glucagon signaling^6,7,54^. Thus, L-Phe and L-Trp are unlikely to play major roles in regulating CaSR in tissues other than in the intestinal tract. Recently, it was reported that inorganic phosphate is a noncompetitive antagonist of CaSR in the parathyroid^40^. However, we did not detect an inhibitory effect of supraphysiological phosphate on αTC1-6 cell proliferation. This could be due to differences in cell types, assay conditions, and/or treatment duration. In addition, phosphate treatment in the study lasted a maximum of 8 h, compared to 5 days in our study^40^.

Our study suggests that CaSR acts through Gq signaling to induce α cell proliferation. CaSR regulates cell proliferation in many tissues^27,50,57^. However, few reports have determined which G protein mediates CaSR action in cell proliferation. Using DREADDs, we tested the 2 major CaSR-activated pathways and found that activation of Gq, but not Gi, induced α cell proliferation (Fig. 5b-e; Supplementary Fig. 5c-f). These results suggest that the Gq pathway may be responsible for CaSR-mediated proliferation. Given the similar actions of Gq and G11 and the known function of G11 in CaSR signal transduction^58,59^, it is possible that G11 may also mediate HAA-activated CaSR signaling in α cells. In addition, we cannot rule out a role for the Gs pathway, which can be activated by CaSR in some cell types^60^. Interestingly, Gq and Gs activation increases β cell mass and proliferation^61,62^, while Gi activation inhibits β cell proliferation^63^. The positive and negative effects on β cell proliferation correlate with the stimulation and inhibition of insulin secretion, respectively. This correlation does not hold true in α cells since several Gi-coupled GPCRs stimulate glucagon secretion^64^. Although Gi activation also increased ERK1/2 phosphorylation, pERK1/2 did not translocate to the nucleus to promote α cell proliferation. The reason for the localization difference is unknown and may be a subject for future investigation. CaSR has also been shown to activate ERK1/2 through β-arrestin^65^. However, β-arrestin-mediated activation of ERK1/2 requires the α subunits of G proteins^66^.

Downstream of the CaSR, Gq is necessary for hyperaminoacidemia-mediated Mek1/2-ERK1/2 activation in α cell proliferation. Mek1/2 phosphorylation of ERK1/2 and subsequent nuclear localization are essential for cell proliferation and are implicated in many cancer types^67,68^. As expected, we found that increased nuclear pERK1/2 correlates with α cell proliferation (Figs. 4a–d,  5, 7) and inhibition of Mek1/2 significantly reduced α cell proliferation both in *gcgr*-deficient zebrafish and in primary mouse islets cultured in high AA medium (Fig. 8). Although it is unsurprising that Mek1/2 activation is required for hyperaminoacidemia-induced α cell proliferation, it is interesting that CaSR is necessary for its activation. We propose that CaSR is a component of the amino acid sensor in α cells that maintains amino acid homeostasis.

A surprising finding is that CaSR is necessary for hyperaminoacidemia-mediated mTORC1 activation. mTORC1 activation is known to be required for hyperaminoacidemia-induced α cell proliferation, and it is thought that increased intracellular AAs caused by AA transporters are the primary driver of mTORC1 activation by hyperaminoacidemia^6–9^. However, we found that CaSR, presumably activated by increased extracellular AAs and acting through Gq-Mek1/2, is also required for mTORC1 activation. A previous study showed that the α1A adrenergic receptor promotes mTORC1 signaling via the Gq pathway in rat-1 fibroblasts^69^. This may result from ERK1/2, PI3K and TSC2 activation^70–72^. Since the *casr* mutants are full-body knockouts, we cannot formally rule out contribution from a non-α cell-autonomous action. The exact mechanism of CaSR-mediated mTORC1 activation in α cells requires further study.

We demonstrated that the Gq and mTORC1 pathways act synergistically to induce α cell proliferation (Fig. 6a–c). Activation individually failed to cause α cell proliferation in *gcgr*-intact fish. However, a high concentration of CNO alone was also sufficient for α cell proliferation in *gcgr*-intact *Tgα*^*Gq*^ fish (Fig. 6d–g). We do not think such a degree of activation is achievable under physiological conditions. In transgenic fish, the Gq-DREADD is under the strongest promoter (*gcga*) in α cells and is likely expressed at much higher levels than endogenous GPCRs. The high agonist concentration would likely achieve supraphysiological levels of Gq activation that is sufficient to turn on both Mek1/2-ERK1/2 and mTORC1 signaling. In contrast, we found that hyperactivation of Gi-DREADD is insufficient for nuclear translocation of pERK1/2 and for mTORC1 activation, explaining its failure to promote α cell proliferation.

In summary, we revealed that activation of mTORC1 and CaSR-Gq-ERK1/2 is necessary and sufficient for α cell proliferation and identified a previously unknown physiological role of CaSR in coupling hyperaminoacidemia to α cell proliferation (Fig. 10i). Using zebrafish and primary mouse islets, the present study demonstrated that the CaSR-Gq-ERK1/2 pathway is another mediator of hyperaminoacidemia-induced α cell proliferation and that CaSR-Gq-Mek1/2 also contributes to mTORC1 activation. Importantly, coactivation of Gq and mTORC1 in α cells causes proliferation independent of hyperaminoacidemia. Future studies should decipher the mechanism of the interplay between Gq-ERK1/2 and mTORC1 during hyperaminoacidemia-induced α cell proliferation. Understanding how hyperaminoacidemia induces α cell proliferation may provide new insights for glucagonoma treatment and for mitigating the undesired effects of the proposed GRA therapy for diabetes.

## Methods

### Ethics statement

The animals were handled in compliance with guidelines and were approved by the Vanderbilt Medical Center Institutional Animal Care and Use Committee (protocol no. M1700143-01 and M2000070-00).

### Zebrafish maintenance

Zebrafish were raised at 28 °C in an Aquatic-Habitats system and embryos were raised at 28.5 °C in an incubator on a 14/10-h light/dark cycle. All animals were staged by days postfertilization (dpf). In this study, *Tg(gcga:EGFP)*^73^ was used to mark α cells for imaging and/or counting α cells. The transgenic and mutant strains used for this study are listed in Supplementary Table 1. The *gcgra*−/−;*gcgrb*−/− fish were described previously^30^.

### Mutagenesis, transgenesis and genotyping

Mutations in *casr* were generated and identified as described^74^. The targeting sequence in sgRNA is GAGTATCTTGACGTCATGG. Two mutant lines (8 bp deletion and 11 bp insertion) in *casr* were characterized for an initial cross-validation before selecting the 8 bp deletion line for all the experiments. To determine *casr* genotypes, tail fin of individual fish was lysed in 20 mM NaOH solution at 95 ^o^C for 20 min. After neutralization by 1 M Tris solution, the DNA was amplified by PCR using a pair of genotyping primers (see supplementary Table 2). The PCR products were then digested with NlaIV (New England Biolabs), which cuts the wild-type product (204 bp) into 94 bp and 110 bp but not the mutant product. The tail fins of larval fish were cut and collected under a dissecting microscope.

Transgenes were generated using the Tol2 System^75^. A 2.8 kb fragment upstream of the ATG of the *gcga* gene was amplified using a pair of primers (see Supplementary Table 2), digested with KpnI/BamHI (New England Biolabs), and cloned into KpnI/BamHI digested p5E-MCS. The resultant p5E-*gcga* was used for α cell specific expression of transgenes. Coding sequences of CaSR, DREADSs, mTOR^CA^, and Rheb^CA^ (see Supplementary Table 1) were amplified using Q5 DNA polymerase (New England Biolabs) and inserted into the pME-MCS vector by traditional cloning or HiFi assembly (New England Biolabs). The CASR-Tango plasmid with codon-optimized human CaSR coding sequence was provided by Brian Roth via Addgene (plasmid #66235). The coding sequence for constitutively active rat Rheb was provided by Mustafa Sahin via Addgene (plasmid # 32520). The coding sequence for constitutively active human mTOR was provided by David Sabatini via Addgene (plasmid # 69006). The hM3Dq and hM4Di plasmids were gifts from Brian Roth (UNC Chapel Hill). Transgenes were coinjected with Tol2 mRNA into zygotes of the desired genetic background and their progeny with germline transmission of the transgenes were identified for the study.

### Free amino acid assay

Free amino acid levels in whole larvae or adult serum were determined using the published method^76^. For larvae, each sample was a homogenate of 40 fish. For adults, each sample is serum from individual tail bleeding. To collect adult blood, fish were anesthetized in 0.02% ice-cold MS-222 (A5040, Sigma) and their tails were cut. Serum was isolated from the blood by centrifugation (1000 g for 15 min) and 10 μL was used. Each sample was homogenized in perchloric acid (weight ratio: 1:50). The homogenate was centrifuged at 15,000 *g* for 10 min. The supernatant was neutralized with 0.8 M KOH (using a volume of KOH 1/19th of the supernatant). After the addition of KOH, the samples were cooled on ice water for approximately 10 min and centrifuged at 15,000 *g* for 10 min. Ten microliters of supernatant was mixed with 3 mL of OPA-MET reagent. After 2–10 min, the fluorescence above the OPA-MET reagent was read in cuvettes on a spectrofluorometer using an excitation wavelength of 340 nm and an emission wavelength of 440 nm. In order to calculate the concentration of the amino acids in the sample, 10 μL of the standard mixture of amino acids (prepared as described above and diluted 1:10, 1:100, and 1:1,000) was mixed with 3 mL of OPA-MET reagent and the fluorescence was read for the sample. The free amino acid levels of larvae were normalized to the total protein concentration, which was measured by the bicinchoninic acid (BCA) assay.

### Proliferation analysis in zebrafish

Proliferation was analyzed using the Click-iT EdU Alexa Fluor 594 Imaging Kit (C10339, Invitrogen). To identify proliferating α cells, 1–2 nl of 0.1 mM 5-ethynyl-2-deoxyuridine (EdU) was injected into the heart at 4 dpf. After 24 h, the fish were euthanized and fixed. EdU was detected according to the modified manufacturer’s instructions. All images were collected using Zeiss LSM780 (Carl Zeiss, Jena, Germany) and analyzed by Imaris (Oxford Instruments, Abingdon, UK) and ImageJ (National Institutes of Health, MD, USA).

### Zebrafish islet isolation, RNA extraction, and qPCR

Larval zebrafish islets were isolated as previously reported^77^. Briefly, *Tg(gcga:EGFP)* larvae were euthanized in ice-cold water and suspended in a solution of HBSS with 50 μg/ml Liberase DH (5401119001, Sigma), lightly crushed with a pestle and incubated at 37 °C for 2 min. The entire content was quickly placed in a 10 cm petri dish containing RPMI (GIBCO) with 10% Fetal Bovine Serum (Atlanta Biologicals). Islets were picked manually under a fluorescence stereomicroscope and placed into a 6 cm dish with RPMI. This process was repeated to limit extraneous tissue. At least 50 islets were pooled for RNA extraction.

RNA of larvae and mouse islets was isolated using TRIZOL reagent (Thermo Fisher Scientific) with Direct-Zol columns (Zymo Research) and concentrated using RNA Clean and Concentrator-25 columns (Zymo Research). RNA was reverse transcribed using Superscript III (Thermo Fisher Scientific) with an oligo-dT primers. The cDNAs were subjected to PCR on a BioRad CFX96 machine (Bio-Rad Laboratories, CA, USA) and amplicons were detected using SYBR green (Bio-Rad). The levels of expression were determined by the Pfaffl method to compare Ct values of *ef1a* (zebrafish) or *Actb* (mouse). All the primers used in this study are listed in Supplementary Table 3.

### Isolation, transduction, and culture of mouse pancreatic islets

All mice used in these studies were 8-18-week-old male C57BL/6J or male and female *Casr*^*flox/flox*^ mice. The experimental mice were maintained in a controlled environment (12-h light/dark cycle, 22 ± 1 °C, 60–70% humidity) with free access to standard chow pellets and water. Mouse pancreata were digested with collagenase P (Roche, Basel, Switzerland), and islets were isolated via density gradient centrifugation as previously described^7^. The islets were then handpicked to >99% purity and cultured in media with high or normal AAs, and with an inhibitor or vehicle for 3 days. The islet culture was based on an RPMI 1640 media formula (GIBCO), except that each AA concentration was individually formulated to mimic the concentrations found in the serum of either wild-type (LAA) or *Gcgr*−/− (HAA) mice^7^ (Supplementary Table 4). RPMI 1640 contains 0.42 mM Ca^2+^ and 5.63 mM of inorganic phosphate. The media was supplemented with 10% fetal bovine serum (Atlanta Biologicals), 1% penicillin/streptomycin (GIBCO), and 5.6 mM D-glucose (Sigma). To inhibit CaSR, 0.5 µM NPS 2143 (Sigma) or 10 µM Calhex 231 (Cayman) was used. To inhibit Mek1/2, 10 µM U0126 (LC laboratories) was used.

For Ad^Cre-GFP^ transduction, the isolated islets were allowed to recover in RPMI 1640 with 10% fetal bovine serum, 1% penicillin/streptomycin, and 5.6 mM D-glucose overnight in the incubator. The next day, they were washed two times with PBS containing 2 mM EDTA and incubated with Accutase for 2 min. The digestion was immediately terminated by the addition of medium with 10% fetal bovine serum. The permeated islets were seeded in a 24-well plate and incubated with Ad^Cre-GFP^ (V1110, Welgen) particles at an MOI of 500. After a 4-h incubation, the islets were washed two times with RPMI 1640 with 10% fetal bovine serum and then transferred into the media with high AA or low AA for 3 days.

After culture, islets were washed in 2 mM EDTA and dispersed in 0.025% Trypsin-2 mM EDTA (GIBCO or HyClone GE Healthcare) for 5–10 min by gentle pipetting to obtain single or very small cell clusters. Dispersed islet cells were recovered by centrifugation in RPMI media containing 5.6 mM D-glucose, 10% fetal bovine serum, and 1% penicillin/streptomycin. The resulting cell pellet was resuspended in 100 μl of media and centrifuged onto a glass slide using a cytospin centrifuge (Thermo Scientific, Waltham, MA, USA). Air-dried slides were stored at −80 °C until use, thawed, and immediately fixed in 4% paraformaldehyde before immunofluorescence. Slides were mounted with Aqua-Poly/Mount (18606, Polysciences).

### Immunofluorescence and imaging

Immunofluorescence on larval zebrafish islet-containing sections was performed as follows: zebrafish larvae were fixed in 4% paraformaldehyde at 5 dpf, equilibrated in 20% sucrose, and embedded into O.C.T. for making cryosections. Islet-containing cryosections (12 μm) were selected for immunofluorescence. Immunofluorescence of cultured mouse islets was performed as described^7^.

The primary antibodies used were anti-pS6 (Ser240/244) (1:300, Cell Signaling Technology, 5364, rabbit), anti-HA-probe (1:200, Santa Cruz Biotechnology, sc-805, rabbit), anti-pERK1/2 (Thr202/Tyr204) (1:150, Cell Signaling Technology, 4370, rabbit), anti-GFP (1:300, Abcam, ab13970, chicken), anti-Glucagon (1:200, Abcam, ab10988, mouse) and anti-Ki67 (1:150, Abcam, ab15580, rabbit or Fisher Scientific, Hu B56, mouse). Secondary antibodies used for visualization were Alexa Fluor 488 (1:1,000, Thermo Fisher Scientific, A-11039, goat anti-chicken), Alexa Fluor 488 (1:1,000, Thermo Fisher Scientific, A-11001, goat anti-mouse), Alexa Fluor 568 (1:1,000, Thermo Fisher Scientific, A-11011, goat anti-rabbit) or Alexa Fluor 647 (1:1,000, Thermo Fisher Scientific, A-21235, goat anti-mouse). Nuclei were stained with Hoechst 33342 (0.5 μg/ml, Thermo Fisher Scientific). The coverslips were mounted on slides with ProLong Diamond mounting medium (Thermo Fisher Scientific). Images were collected using a Zeiss LSM880 (Carl Zeiss, Jena, Germany) and analyzed by Imaris (Oxford Instruments, Abingdon, UK) and ImageJ (National Institutes of Health, MD, USA).

### Effect of phosphate, glutamine, and Ca^2+^ on αTC1-6 cells

We used two kits to measure αTC1-6 cells in 96-well plates: the Promega CellTiter 96® AQueous One Solution Cell Proliferation Assay (Promega, 3580) and the CyQuant cell proliferation kit (Thermo Fisher Scientific, C7026). For the Promega kit, the culture started at 8000 cells/well. For the Thermo kit, 5000 cells/well were used. The basal medium was MEM (no calcium and no glutamine, GIBCO) supplemented with 5% (v/v) fetal bovine serum (Atlanta Biologicals), 10 mM HEPES (GIBCO), and 1% penicillin/streptomycin (GIBCO)^6^. To determine if increased glutamine increases αTC1-6 cell proliferation, Ca^2+^ (CaCl_2_, Sigma) was added to 1.5 mM, and glutamine (Sigma) was added to 1, 2, 3, 4, or 5 mM. Each condition was repeated in eight wells. To determine if Ca^2+^ affects α cell proliferation, glutamine (Sigma) was added to 4 mM and Ca^2+^ (CaCl_2_, Sigma) was added to 0.5, 1, 1.5, 2, or 2.5 mM. Each condition was repeated in eight wells. The relative cell number was determined at day 5 using the Promega kit according to the manufacturer’s instructions. Values were corrected for background absorbance using the average of the cell-free wells containing assay medium. To determine the role of CaSR in αTC1-6 proliferation, cells were cultured for 5 days in the proliferation medium (basal medium plus 1.5 mM Ca^2+^ and 4 mM glutamine) in the presence or absence of 2 µM NPS 2143. Each condition was repeated in ten wells. To determine the effects of phosphate concentrations, αTC1-6 cells were cultured in a 96-well plate for 5 days in the proliferation medium with 1.2 mM or 5.6 mM phosphate. Each condition was repeated in 10 wells. On day 4, a 2× serial dilution of αTC1-6 cells from 20,000/100 µl to 625/100 µl was made. One hundred microliters from each dilution was seeded in the empty wells in triplicate and cultured in basal medium with 1.5 mM Ca^2+^ overnight to serve as cell number standards. The cell number was measured using the Thermo kit.

### Ca^2+^ concentration assay, alizarin red S (ARS) staining, and micro-CT

The Ca^2+^ concentration was measured by ICP-MS. Briefly, 1.5 mL polypropylene microcentrifuge tubes (Thermo Fisher Scientific) were immersed and washed three times in 10% nitric acid (67–69%, Optima™; Thermo Fisher Scientific) overnight. After acid treatment, the tubes were rinsed thoroughly with ultrapure water (Processed by Milli-Q™). Afterward, embryos (7 dpf) were washed 3 times with cold ultrapure water, and four 20-fish pools were placed in tubes for each group. For metal measurement, 200 μL nitric acid was added to digest samples at 85 °C until dry. The samples were then suspended in nitric acid and dried again. Two hundred microliters of hydrogen peroxide (30%, Optima™; Thermo Fisher Scientific) was added and evaporated at 85 °C twice. Eventually, the samples were diluted in 1.5 mL 2% nitric acid. Empty tubes, processed identically as tubes containing embryos, work as mock treatments. The Ca^2+^ level was normalized to sulfur, and the standard curves were generated to measure the content of Ca^2+^ by Agilent 7700 series ICP-MS (Agilent Technologies).

ARS staining was used to measure larval zebrafish skeletal mineralization. Briefly, zebrafish were fixed in 4% PFA at 10 dpf and then stained for 15 min with 0.01 % ARS (3,4-dihydroxy-9,10-dioxo-2-anthracenesulfonic acid sodium salt, Sigma) dissolved in 70% ethanol. After rinsing three times/5 min in PBS, the samples were imaged immediately under 510 nm excitation fluorescence with Zeiss LSM780 (Carl Zeiss, Jena, Germany) and analyzed by Imaris 9.5.1 (Oxford Instruments, Abingdon, UK) and ImageJ 1.49 (National Institutes of Health, MD, USA).

Three zebrafish (60 dpf) were sacrificed for each group and kept in 75% alcohol at −20 °C until use. The sample was then placed in a sample holder in which a sponge was used to fix the sample. Micro-CT scanning was run via μCT 50 (Scanco Medical, Switzerland). High-resolution scans (5 μm voxel resolution) were obtained using the following parameters: X-ray energy 45 kVp, current 88 μA, sampling rate 2048, 1000 proj/180°, integration time 300 ms, which were modified according to the previously published methods^78^. The micro-CT images were processed using custom software to generate the bone structures. The total volume (TV), bone volume (BV), bone volume fraction (BV/TV), and tissue mineral density were calculated.

### Chemical treatment in zebrafish experiment

All drugs were made in 1000 X stock solution and stored in light-protected Eppendorf tubes at −20 °C. CNO (20 mmol/L; Tocris), NPS 2143 (20 mmol/L; Sigma), U0126 (10 mmol/L; LC laboratories), and rapamycin (2.5 mmol/L; Cayman) were dissolved in DMSO at the indicated concentrations. Zebrafish were exposed to 1 μM or 20 μM CNO, 1 μM NPS 2143, 10 μM U0126, and 2.5 µM rapamycin for 2 days, respectively, starting at 3 dpf and injected with EdU at 4 dpf. All drug stocks were dissolved in egg water within a petri dish (10 cm) to the working concentration. Larvae were fixed and stained at 5 dpf for α cell number and proliferation assays.

### Statistical analysis

For group comparisons, either one-way analysis of variance with Tukey’s honest significant difference (HSD) post hoc test or two-tailed unpaired Student’s *t* test was used. A *P* value less than 0.05 was considered statistically significant. Values represent means ± SD. Analyses were performed using GraphPad Prism 8.0.1 (GraphPad Software, San Diego, CA).

### Reporting summary

Further information on research design is available in the Nature Portfolio Reporting Summary linked to this article.

## Supplementary information

Supplementary Information

Reporting Summary

## Data Availability

The authors declare that all data supporting the findings of this study are available within the article, the source data provided with this paper, and the supplementary information, or from the corresponding author upon request without restrictions. Source data are provided with this paper.

## Source data

Source Data Please use the attached new Source data file.
