# Supplementary material for: Hyperaminoacidemia induces pancreatic α cell proliferation via synergism between the mTORC1 and CaSR-Gq signaling pathways
Source: Nat Commun. 2023 Jan 16;14:235. doi: 10.1038/s41467-022-35705-4 (PMC9842633; doi:10.1038/s41467-022-35705-4)
Supplement: Supplementary file 1 — Supplementary Information [file 41467_2022_35705_MOESM1_ESM.pdf]

Supplementary Figure 1 Constitutive activation of mTORC1 increases α cell area and free amino acids level in adult zebrafish.

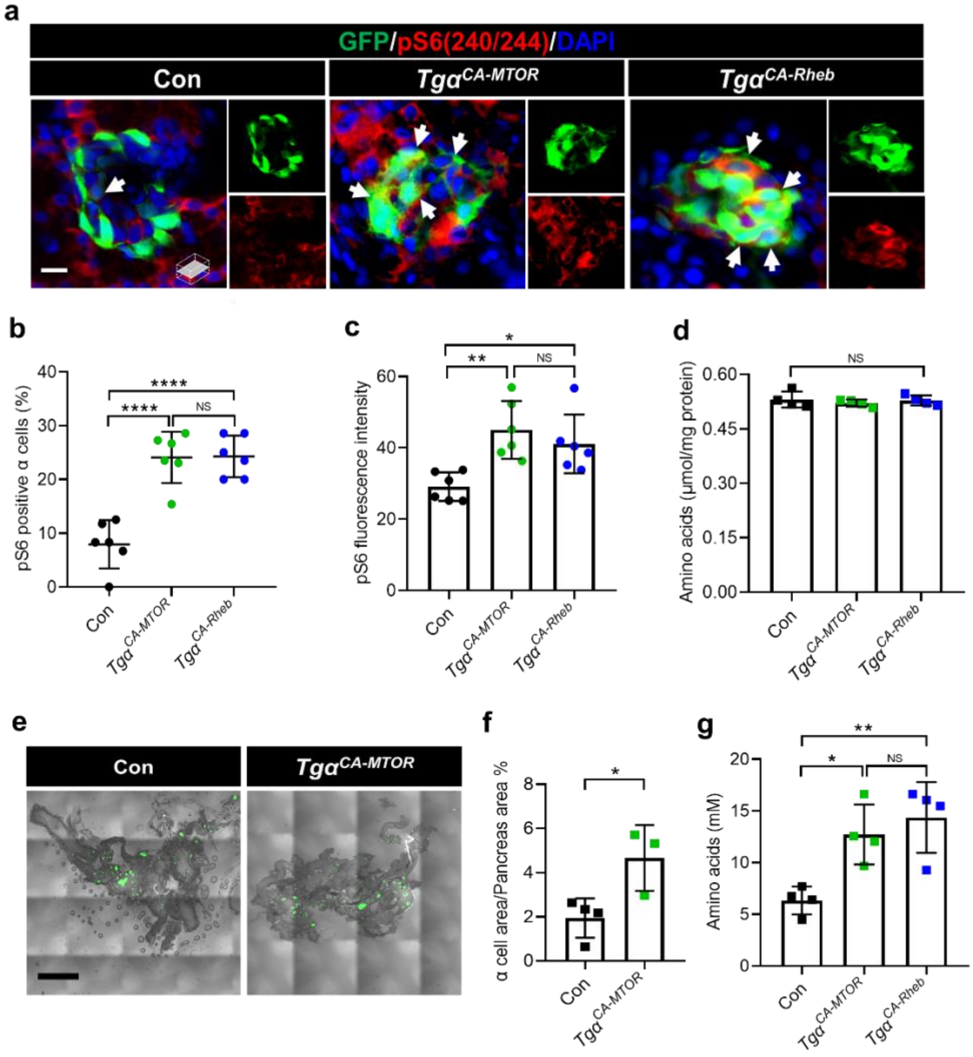

S Fig. 1 Constitutive activation of mTORC1 increases α cell area and free amino acid levels in adult zebrafish. a. Representative images of pS6(240/244) and GFP immunofluorescence in islet sections of control, *Tgα*$^{CA\text{-}MTOR}$ and *Tgα*$^{CA\text{-}Rheb}$ larvae at 5 dpf. pS6(240/244) signal in α cell is indicated by arrows, primary antibody: anti-pS6 (Ser240/244) (1:300, rabbit); secondary antibody: Alexa Fluor 568 (1:1,000, goat anti-rabbit) (scale bar, 8 μm). **b.** Quantification of the percentages of pS6(240/244) positive α cells in control, *Tgα*$^{CA\text{-}MTOR}$ and *Tgα*$^{CA\text{-}Rheb}$ larvae at 5 dpf (data represent the means ± SD, n = 6 for each group). **c.** Quantification of the pS6(240/244) fluorescence intensity in α cells of control, *Tgα*$^{CA\text{-}MTOR}$ and *Tgα*$^{CA\text{-}Rheb}$ larvae at 5 dpf (data represent the means ± SD, n = 6 for each group). **d.** Free amino acids levels of control, *Tgα*$^{CA\text{-}MTOR}$ and *Tgα*$^{CA\text{-}Rheb}$ larvae at 5 dpf, normalized by total protein concentration (data represent the means ± SD, n = 4, 40 fish/replicate, for each group). **e**. Representative images of whole-mount adult pancreas of control and *Tgα*$^{CA\text{-}MTOR}$ zebrafish showing the α cell areas (scale bar, 1000 μm). **f**. Quantification of the percentages of area occupied by α cells in pancreas of control and *Tgα*$^{CA\text{-}MTOR}$ zebrafish adults (data represent the means ± SD, n = 4 and 3 for each group, respectively). **g.** Serum free amino acid levels of control, *Tgα*$^{CA\text{-}MTOR}$ and *Tgα*$^{CA\text{-}Rheb}$ zebrafish adults (data represent the means ± SD, n = 4 for each group). **P* < 0.05, ***P* < 0.01, *****P* < 0.0001, NS indiates no significant difference (one-way ANOVA, Tukey's multiple comparisons test for S Fig. 2b, c, d and g; two-tailed unpaired t test for S Fig. 2f; the quantifications represent individual islet sections). Source data are provided as a Source Data file.

Supplementary Figure 2 Validation of knockdown by *casr* sgRNA, *gprc6a* sgRNA and *tas1r3* sgRNA in *gcgr*^{DKO} zebrafish.

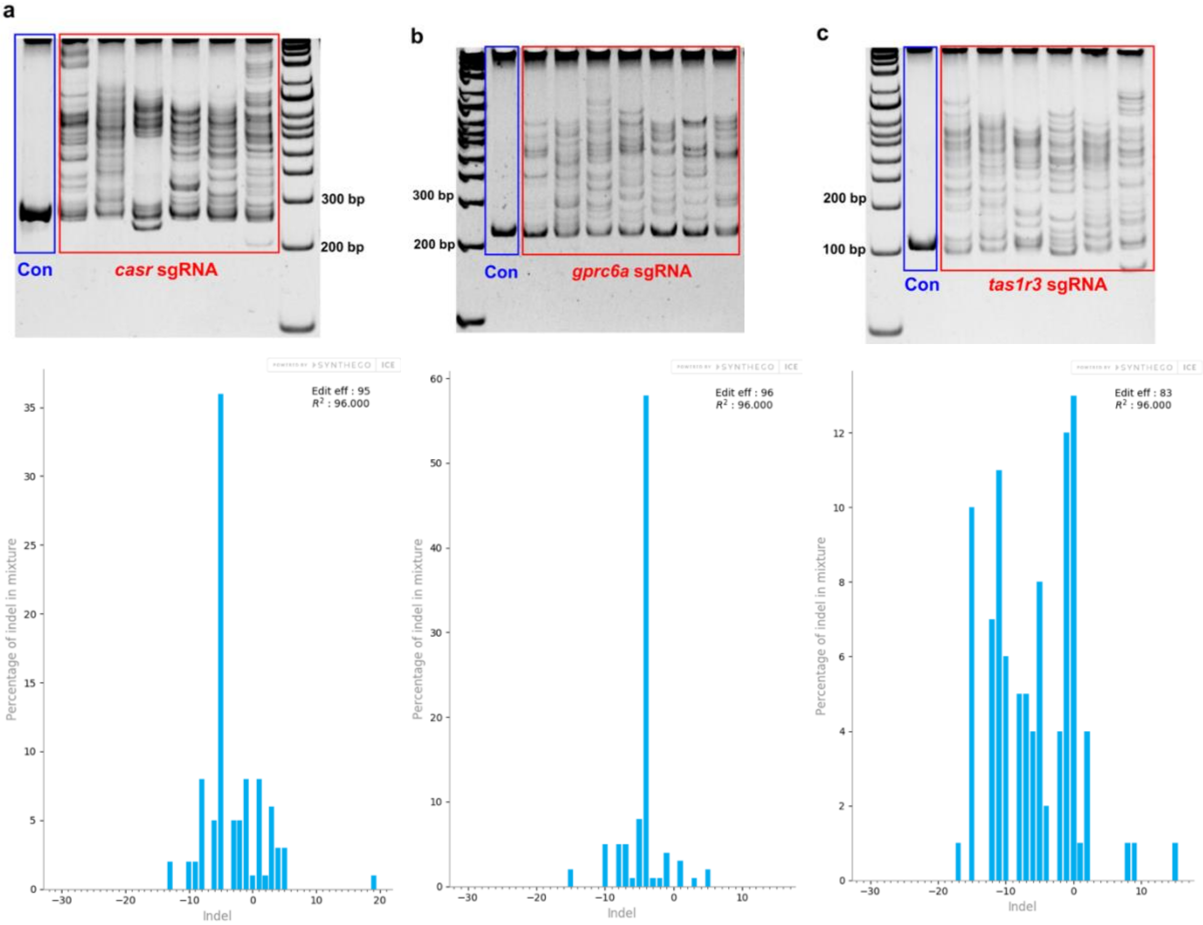

S Fig. 2 Assessment of knockdown efficiency by *casr* sgRNA, *gprc6a* sgRNA and *tas1r3* sgRNA in *gcgr*^{DKO} zebrafish. a. Gel image of heteroduplex motility assay (HMA) of the *casr* sgRNA mutated fish (top) and indel distribution histogram from Synthego ICE analysis (bottom). **b.** HMA image of the *gprc6a* sgRNA mutated fish (top) and indel distribution histogram from Synthego ICE analysis (bottom). **c.** HMA image of the *tas1r3* sgRNA mutated fish (top) and indel distribution histogram from Synthego ICE analysis (bottom). Primers flanking the sgRNA target in each gene were used to amplify the genomic DNA from individual mutated fish. Mutated fish are genetically mosaic at the targeted locus and may differ between individuals. As such, the PCR products of the target region are highly heterogenous, consist of both residual wildtype and numerous mutant sequences. When these sequences reanneal after denaturation, heteroduplexes form. When the reannealed products were run in a native PAGE gel, the numerous species of heteroduplexes migrate at various rates, always slower than homoduplexes due to the structure deformation. Therefore, the amount of slower migrating species is positively correlated to the efficiency of mutagenesis. The products at the position of WT from the mutated fish consists of homoduplexes of both WT and mutants. The products smaller than the WT are probably homoduplexes of large deletions. For ICE analysis, the target region was amplified from genomic DNA of a pool of 20 one-day-old WT or Cas9-sgRNA injected embryos. The PCR products were individually sequenced using Sanger method. The .ab1 files were submitted for ICE analysis (https://ice.synthego.com/#/).

Supplementary Figure 3 Loss of *casr* results in hypercalcemia, skeletal dysplasia and growth retardation.

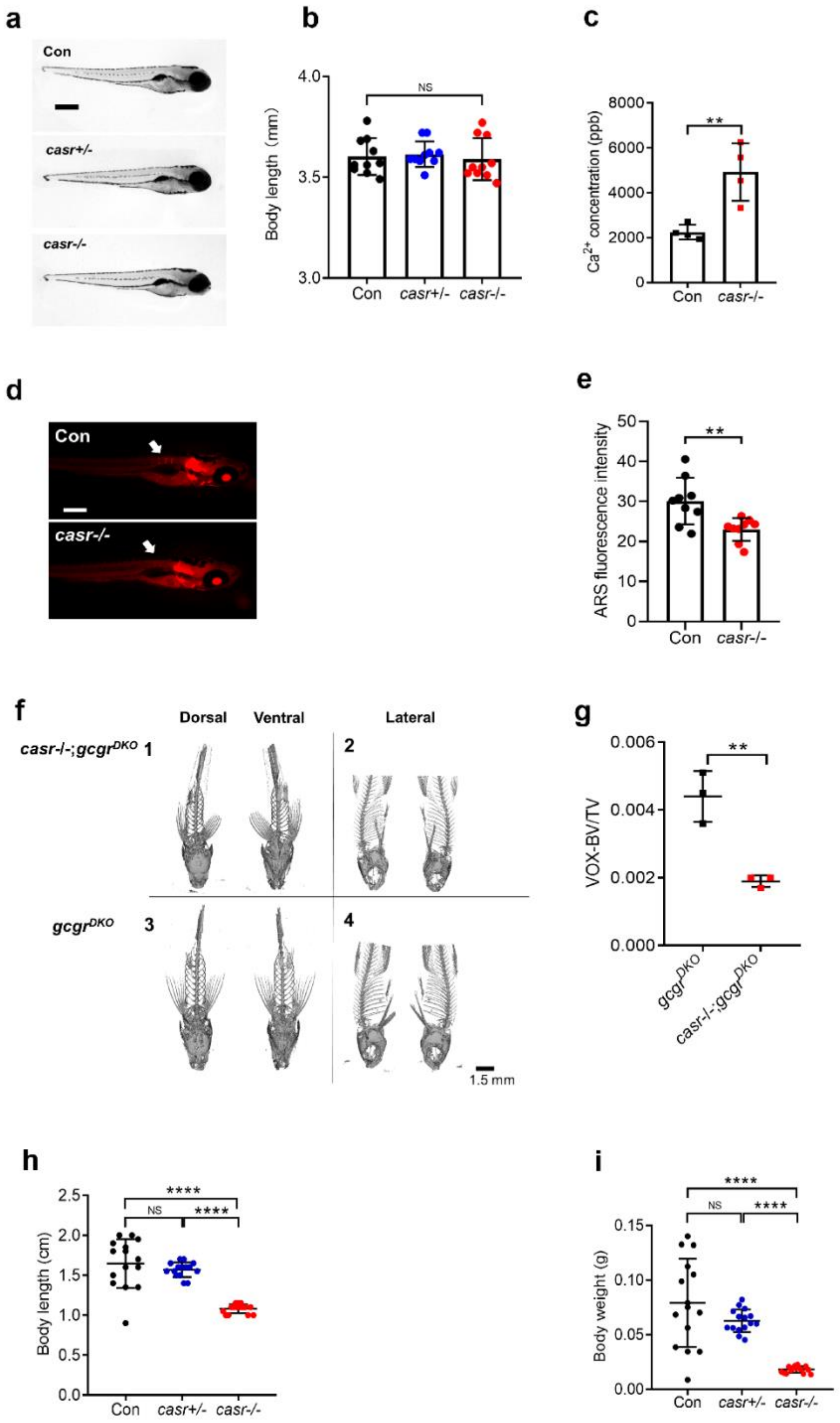

S Fig. 3 Loss of *casr* results in hypercalcemia, skeletal dysplasia and growth retardation. a. Gross appearance of control, *casr* +/- and *casr-/-* larvae at 5 dpf (scale bar, 500 μm). **b.** The body length of control, *casr* +/- and *casr-/-* larvae at 5 dpf (data represent the means ± SD, n = 10 for each group). **c.** Body Ca^{2+} concentration of control and *casr-/-* larvae at 7 dpf (data represent the means ± SD, n = 4, 20 fish/replicate, for each group). **d.** Representative images of alizarin red S (ARS) staining in control and *casr-/-* larvae at 10 dpf. Calcium deposition in notochord is indicated by arrows (scale bar, 300 μm). **e.** Quantification of ARS fluorescence intensity in the notochord of control and *casr-/-* larvae at 10 dpf (data represent the means ± SD, n = 9 for each group). **f.** Representative images of micro-CT scan in $gcgr^{DKO}$ and *casr*-/-;$gcgr^{DKO}$ zebrafish at 2 months of age. **g.** Quantification of bone volume fraction (Bone volume/Body volume) in micro-CT graph (data represent the means ± SD, n = 3 for each group). **h.** Body length of zebrafish at 2 months of age (data represent the means ± SD, n = 15 for each group) **i.** Body weight of zebrafish at 2 months of age (data represent the means ± SD, n = 15 for each group). **$P < 0.01$, ****$P < 0.0001$, NS indicates no significant difference (one-way ANOVA, Tukey's multiple comparisons test for S Fig. 5. b, h and i; two-tailed unpaired t test for S Fig. 5. c, e and g). Source data are provided as a Source Data file.

Supplementary Figure 4 CaSR is essential and glutamine-dependent for α cell proliferation in zebrafish and mouse islet.

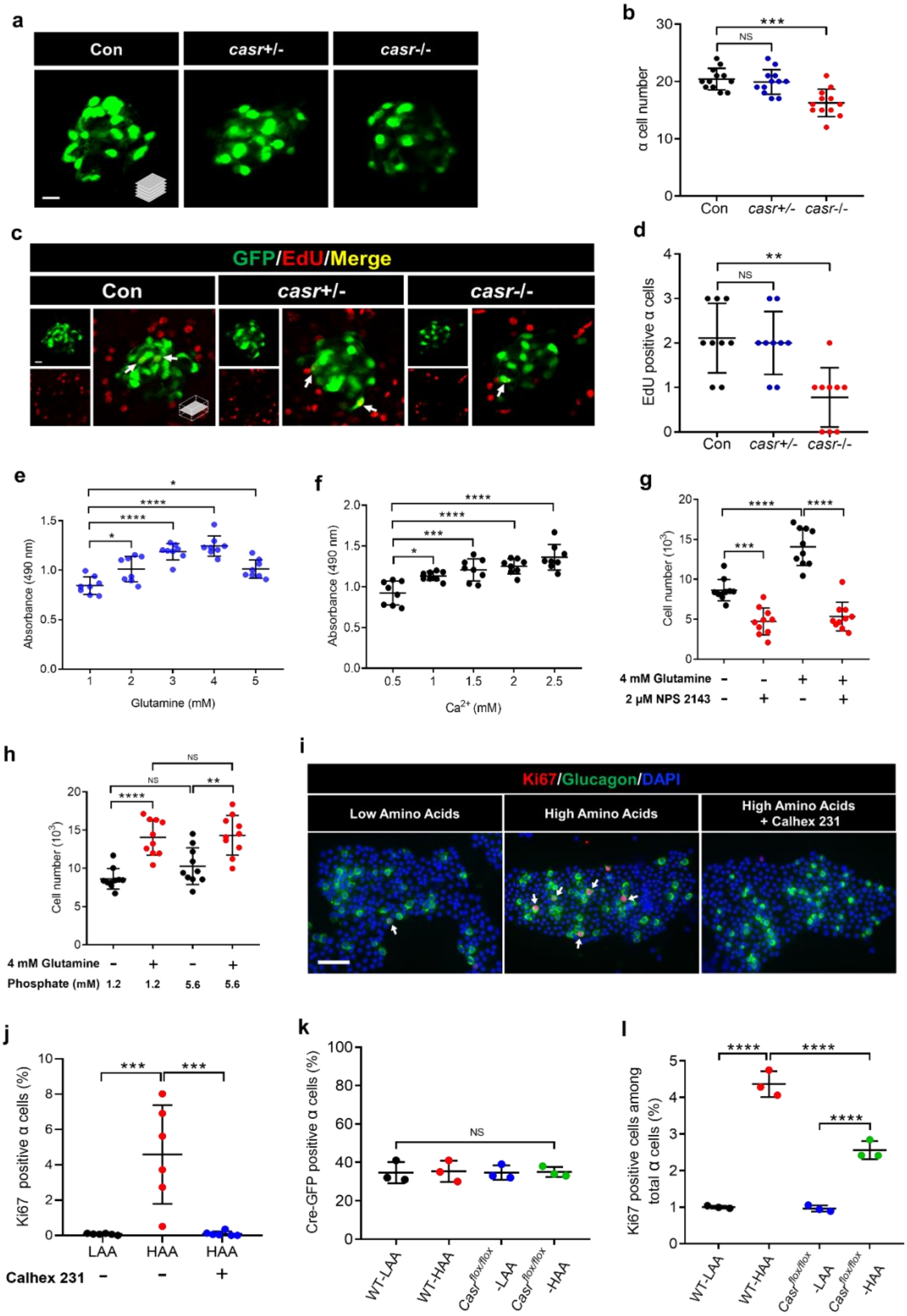

S Fig. 4 CaSR is essential and glutamine-dependent for α cell proliferation in zebrafish and mouse islet. a. Representative images of α cells in control, *casr* +/- and *casr-/-* larvae at 5 dpf (scale bar, 10 μm). **b.** Quantification of the α cell numbers in control, *casr* +/- and *casr-/-* larvae at 5 dpf (data represent the means ± SD, n = 12 for each group). **c.** Representative images of EdU staining in control, *casr* +/- and *casr-/-* larvae at 5 dpf. EdU (red)-positive α cells is indicated by arrows (scale bar, 10 μm). **d.** Quantification of EdU positive α cell numbers in control, *casr* +/- and *casr-/-* larvae at 5 dpf (data represent the means ± SD, n = 9 for each group). **e.** Relative αTC1-6 cell numbers cultured in 1.5 mM Ca^{2+} and different concentrations of glutamine for 5 days (data represent the means ± SD, n = 8 for each group). **f.** Relative αTC1-6 cell numbers cultured in 4 mM glutamine and different concentrations of Ca^{2+} for 5 days (data represent the means ± SD, n = 8 for each group). **g.** The αTC1-6 cell numbers cultured with 2 μM NPS 2143 or vehicle in the presence or absence of 4 mM glutamine for 5 days (data represent the means ± SD, n = 10 for each group). **h.** The αTC1-6 cell numbers cultured with 4 mM glutamine or vehicle with 1.2 mM or 5.6 mM phosphate for 5 days (data represent the means ± SD, n = 10 for each group). **i.** Representative images of cells from primary mouse islets cultured with LAA, HAA, and HAA + Calhex 231 (10 μM) media. Ki67(red)-positive α cells are indicated by arrows, primary antibody: anti-Glucagon (1:200, mouse)/anti-Ki67 (1:150, rabbit); secondary antibody: Alexa Fluor 488 (1:1,000, goat anti-mouse)/Alexa Fluor 568 (1:1,000, goat anti-rabbit) (scale bar, 50 μm). **j.** Quantification of the percentages of Ki67-positive α cells in mouse islets cultured with LAA, HAA, and HAA + Calhex 231 (10 μM) media (data represent the means ± SD, n = 6 for each group). **k.** Quantification of the percentages of Cre-GFP positive α cells in WT and $Casr^{flox/flox}$ mouse islets cultured with LAA and HAA after $Ad^{Cre\text{-}GFP}$ transduction (data represent the means ± SD, n = 3 for each group). **l.** Quantification of the percentages of Ki67-positive cells among total α cells of WT and $Casr^{flox/flox}$ mouse islets cultured with LAA and HAA media after $Ad^{Cre\text{-}GFP}$ transduction (data represent the means ± SD, n = 3 for each group). $^{*}P < 0.05$, $^{**}P < 0.01$, $^{***}P < 0.001$, $^{****}P < 0.0001$, NS indicates no significant difference (one-way ANOVA, Tukey's multiple comparisons test, the quantifications represent individual islet sections). Source data are provided as a Source Data file.

Supplementary Figure 5 Activation of a Gi-DREADD in α cells fails to restore proliferation in *casr-/-*;*gcgr*^{DKO} zebrafish.

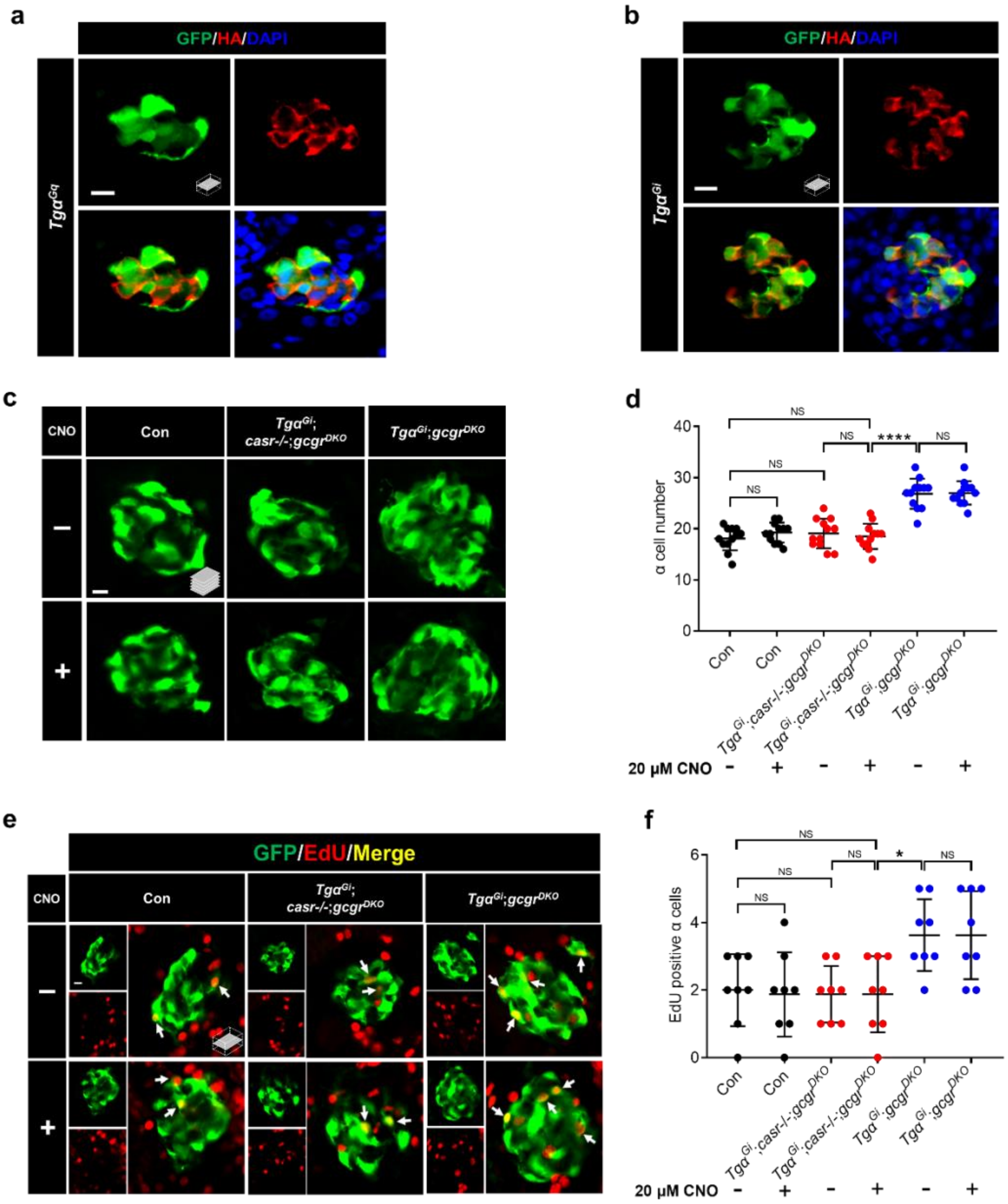

S Fig. 5 Activation of a Gi-DREADD (hM4Di) in α cells fails to restore proliferation in *casr-/-*;*gcgr*^{DKO} zebrafish. a. Representative images of HA and GFP immunofluorescence in islet sections of *Tgα*^{Gq} at 5 dpf, primary antibody: anti-HA-probe (1:200, rabbit); secondary antibody: Alexa Fluor 568 (1:1,000, goat anti-rabbit) (scale bar, 10 μm). **b.** Representative images of HA and GFP immunofluorescence in islet sections of *Tgα*^{Gi} at 5 dpf, primary antibody: anti-HA-probe (1:200, rabbit); secondary antibody: Alexa Fluor 568 (1:1,000, goat anti-rabbit) (scale bar, 10 μm). **c.** Representative images of α cells in control, *Tgα*^{Gi};*casr*-/-;*gcgr*^{DKO} and *Tgα*^{Gi};*gcgr*^{DKO} larvae treated with 20 μM CNO or vehicle for 48 h at 5 dpf (scale bar, 10 μm). **d.** Quantification of α cell numbers in control, *Tgα*^{Gi};*gcgr*^{DKO} and *Tgα*^{Gi} *casr*-/-;*gcgr*^{DKO} larvae treated with 20 μM CNO or vehicle for 48 h at 5 dpf (data represent the means ± SD, n = 12 for each group). **e.** Representative images of EdU staining in control, *Tgα*^{Gi};*casr*-/-;*gcgr*^{DKO} and *Tgα*^{Gi};*gcgr*^{DKO} larvae treated with 20 μM CNO or vehicle for 48 h at 5 dpf. EdU (red)-positive α cells are indicated by arrows (scale bar, 10 μm). **f.** Quantification of EdU positive α cell numbers in control, *Tgα*^{Gi};*casr*-/-;*gcgr*^{DKO} and *Tgα*^{Gi};*gcgr*^{DKO} larvae treated with 20 μM CNO or vehicle for 48 h at 5 dpf (data represent the means ± SD, n = 8 for each group). *$P < 0.05$, ****$P < 0.0001$, NS indicates no significant difference (one-way ANOVA, Tukey's multiple comparisons test, the quantifications represent individual islet sections). Source data are provided as a Source Data file.

Supplementary Figure 6 Synergism of the Gq and mTORC1 pathways is sufficient to elevate islet *gcga*/*gcgb* expression.

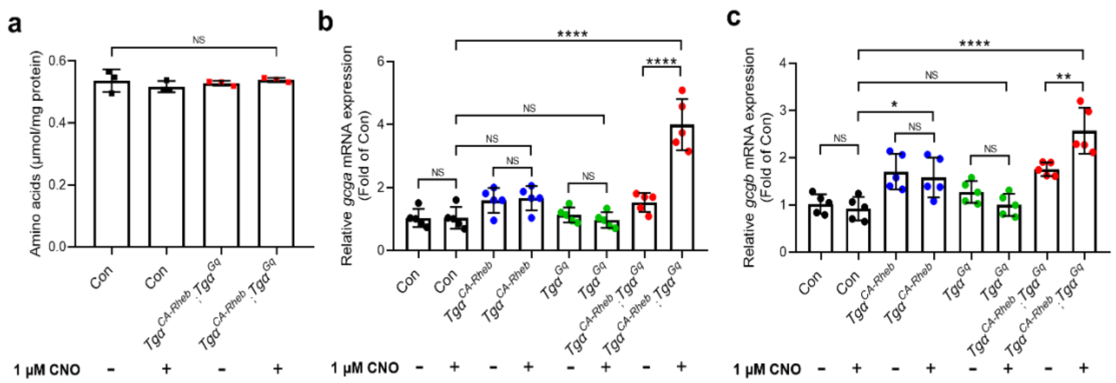

S Fig. 6 Synergism of the Gq and mTORC1 pathways is sufficient to elevate islet *gcga*/*gcgb* expression. a. Free amino acids levels of control and $Tg\alpha^{CA\text{-}Rheb}$;$Tg\alpha^{Gq}$ larvae treated with 1 μM CNO or vehicle for 48 h at 5 dpf, normalized by total protein concentration (data represent the means ± SD, n = 3, 40 fish/replicate, for each group). **b.** qPCR analysis of islet *gcga* in control, $Tg\alpha^{CA\text{-}Rheb}$, $Tg\alpha^{Gq}$ and $Tg\alpha^{CA\text{-}Rheb}$;$Tg\alpha^{Gq}$ larvae treated with 1 μM CNO or vehicle for 48 h at 5 dpf (data represent the means ± SD, n = 5 for each group). **c.** qPCR analysis of islet *gcgb* in control, $Tg\alpha^{CA\text{-}Rheb}$, $Tg\alpha^{Gq}$ and $Tg\alpha^{CA\text{-}Rheb}$;$Tg\alpha^{Gq}$ larvae treated with 1 μM CNO or vehicle for 48 h at 5 dpf (data represent the means ± SD, n = 5 for each group). $^{*}P < 0.05$, $^{**}P < 0.01$, $^{****}P < 0.0001$, NS indicates no significant difference (one-way ANOVA, Tukey's multiple comparisons test). Source data are provided as a Source Data file.

Supplementary Figure 7 Activation of a Gi-DREADD in α cells fails to fully restore ERK1/2 activation in *casr-/-*;*gcgr*^{DKO} zebrafish.

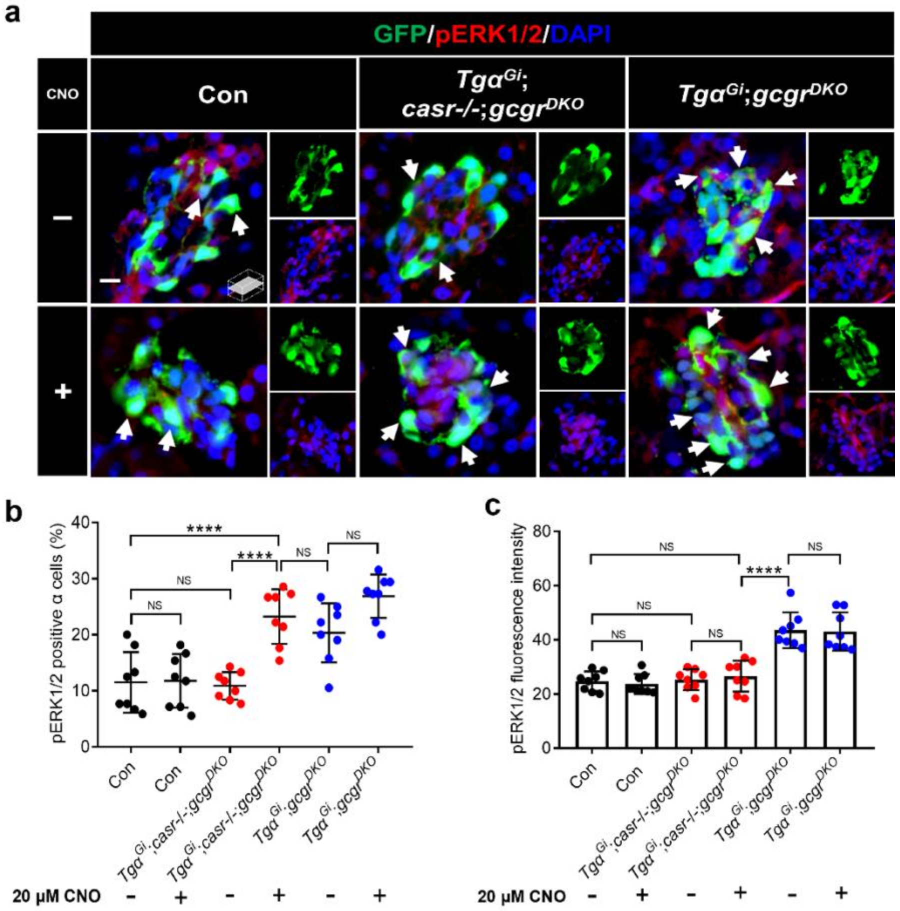

S Fig. 7 Activation of a Gi-DREADD in α cells fails to fully restore ERK1/2 activation in *casr-/-;gcgr^{DKO}* zebrafish. a. Representative images of pERK1/2 and GFP immunofluorescence in islet sections of control, *Tgα^{Gi};casr-/-;gcgr^{DKO}* and *Tgα^{Gi};gcgr^{DKO}* larvae treated with 20 μM CNO or vehicle for 48 h at 5 dpf. The pERK1/2 signal in α cell is indicated by arrows, primary antibody: anti-pERK1/2 (Thr202/Tyr204) (1:150, rabbit); secondary antibody: Alexa Fluor 568 (1:1,000, goat anti-rabbit) (scale bar, 8 μm). **b.** Quantification of the percentage of the pERK1/2 positive α cells in control, *Tgα^{Gi}; casr-/-;gcgr^{DKO}* and *Tgα^{Gi};gcgr^{DKO}* larvae treated with 20 μM CNO or vehicle for 48 h at 5 dpf (data represent the means ± SD, n = 8 for each group). **c.** Quantification of the pERK1/2 fluorescence intensity in α cells of control, *Tgα^{Gi};casr-/-;gcgr^{DKO}* and *Tgα^{Gi};gcgr^{DKO}* larvae treated with 20 μM CNO or vehicle for 48 h at 5 dpf (data represent the means ± SD, n = 8 for each group). ****P < 0.0001, NS indicates no significant difference (one-way ANOVA, Tukey's multiple comparisons test, the quantifications represent individual islet sections). Source data are provided as a Source Data file.

Supplementary Figure 8 Activation of a Gi-DREADD in α cells fails to restore mTORC1 activation in *casr-/-*;*gcgr*^{DKO} zebrafish.

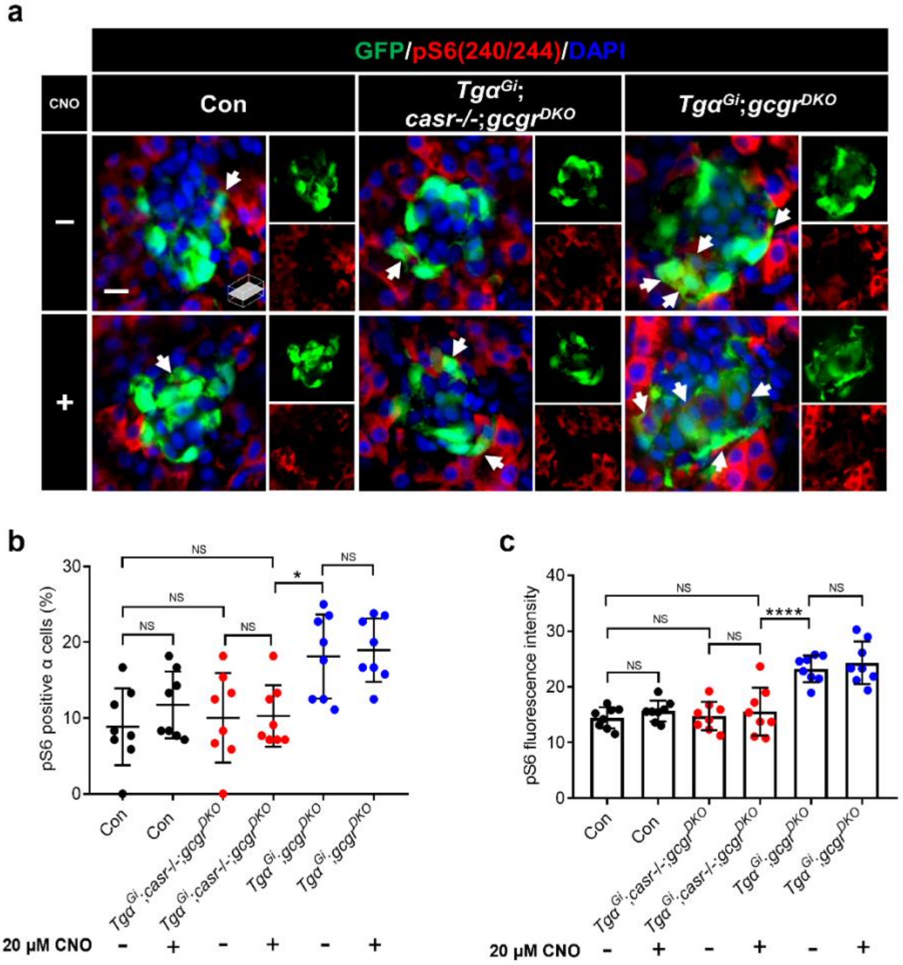

S Fig. 8 Activation of a Gi-DREADD in α cells fails to restore mTORC1 activation in *casr-/-;gcgr*^{DKO} zebrafish. a. Representative images of pS6(240/244) and GFP immunofluorescence in islet sections of control, *Tgα*^{Gi};*casr-/-;gcgr*^{DKO} and *Tgα*^{Gi};*gcgr*^{DKO} larvae treated with 20 μM CNO or vehicle for 48 h at 5 dpf. The pS6(240/244) signal in α cell is indicated by arrows, primary antibody: anti-pS6 (Ser240/244) (1:300, rabbit); secondary antibody: Alexa Fluor 568 (1:1,000, goat anti-rabbit) (scale bar, 7 μm). **b.** Quantification of the pS6(240/244) positive α cell percentages in control, *Tgα*^{Gi};*casr-/-;gcgr*^{DKO} and *Tgα*^{Gi};*gcgr*^{DKO} larvae treated with 20 μM CNO or vehicle at 5 dpf (data represent the means ± SD, n = 8 for each group). **c.** Quantitative results of the pS6(240/244) fluorescence intensity in control, *Tgα*^{Gi};*casr-/-;gcgr*^{DKO} and *Tgα*^{Gi};*gcgr*^{DKO} larvae α cell treated with 20 μM CNO or vehicle at 5 dpf (data represent the means ± SD, n = 8 for each group). *$P < 0.05$, ****$P < 0.0001$, NS indicates no significant difference (one-way ANOVA, Tukey's multiple comparisons test, the quantifications represent individual islet sections). Source data are provided as a Source Data file.

Supplementary Table 1 Transgenic and mutant strains

Abbreviation	Mutant lines[1]
Con	Wild Type
gcgr^{DKO}	*gcgra-/-;gcgrb-/-*
Tgα^{CaSR}	*Tg(gcga:CaSR, cryaa:tagRFP)*
Tgα^{Gq}	*Tg(gcga:hM3Dq, cryaa:tagRFP)*
Tgα^{Gi}	*Tg(gcga:hM4Di, cryaa:tagRFP)*
Tgα$^{CA\text{-}MTOR}$	*Tg(gcga:MTOR*^{L1460P}*, cryaa:tagRFP)*
Tgα$^{CA\text{-}Rheb}$	*Tg(gcga:Rheb*^{S16H}*, cryaa:YFP)*

1 All the mutant lines are in the *Tg(gcga:EGFP)* background.

Supplementary Table 2 List of primers used in *casr* mutant genotyping and *gcga* promoter amplifying

Gene name	Forward primer	Reverse primer
genotyping	ATGGTGAGACGAAACATCACAG	TTCTCGCACAAATTCATTATGG
amplifying[1]	gaggtaccAAGCGATTGTTATGAGTTCCTGAG	caaggatcCAGCTGAGTCTTCCAACACACACC

1 The lowercases are added RE sites for cloning into p5E-MCS.

Supplementary Table 3 List of primers used in qPCR analysis

Gene name	Forward primer	Reverse primer
casr	AAATGCCCAAACAACTCCTG	GGTTTGATGCCTTCACGATT
tas1r3	AAGCCAACGTGAGCATGGTA	ATATGCGGCACAGTAGACGG
gprc6a	CCGAAACGGAGGGAATTTGC	TATCACTGGCAACCCACACC
gcga	AAATGCATTTGCGTCCCACTGC	CATGAGCCACTGAACGAAGTCTTGT
gcgb	GGAAAACGGCAGCCTTATGTCTG	CGTGTCGGGACTCCACTCCTCT
eifa	CTTCTCAGGCTGACTGTGC	CCGCTAGCATTACCCTCC
Slc38a5	TCATCATCTGTCTGCACAAACG	TCAAGAACCAGTCTCCCTCA
Actb	GATTACTGCTCTGGCTCCTAG	GACTCATCGTACTCCTGCTTG

Supplementary Table 4. AA composition of HAA and LAA media (in µM)

Amino acids (µM)	LAA	HAA
Glycine	200	1500
L-Arginine	40	600
L-Asparagine	40	350
L-Aspartic acid	10	10
L-Cystine 2HCl	10	10
L-Glutamic Acid	100	300
L-Glutamine	500	3250
L-Histidine	40	250
L-Hydroxyproline	0	0
L-Isoleucine	125	250
L-Leucine	225	400
L-Lysine hydrochloride	200	1200
L-Methionine	60	280
L-Phenylalanine	75	75
L-Proline	85	400
L-Serine	100	1250
L-Threonine	150	1750
L-Tryptophan	65	65
L-Tyrosine	50	250
L-Valine	300	600
Alanine	350	2250
Ornithine	100	400
