# Supplementary material for: Hyperaminoacidemia induces pancreatic α cell proliferation via synergism between the mTORC1 and CaSR-Gq signaling pathways
Source: Nat Commun. 2023 Jan 16;14:235. doi: 10.1038/s41467-022-35705-4 (PMC9842633; doi:10.1038/s41467-022-35705-4)
Supplement: Supplementary file 2 — Reporting Summary [file 41467_2022_35705_MOESM2_ESM.pdf]

nature portfolio

Corresponding author(s): Wenbiao Chen

Last updated by author(s): Dec 5, 2022

Reporting Summary

Nature Portfolio wishes to improve the reproducibility of the work that we publish. This form provides structure for consistency and transparency in reporting. For further information on Nature Portfolio policies, see our Editorial Policies and the Editorial Policy Checklist.

Statistics

For all statistical analyses, confirm that the following items are present in the figure legend, table legend, main text, or Methods section.

n/a	Confirmed	
☐	☒	The exact sample size (*n*) for each experimental group/condition, given as a discrete number and unit of measurement
☐	☒	A statement on whether measurements were taken from distinct samples or whether the same sample was measured repeatedly
☐	☒	The statistical test(s) used AND whether they are one- or two-sided<br>*Only common tests should be described solely by name; describe more complex techniques in the Methods section.*
☒	☐	A description of all covariates tested
☐	☒	A description of any assumptions or corrections, such as tests of normality and adjustment for multiple comparisons
☐	☒	A full description of the statistical parameters including central tendency (e.g. means) or other basic estimates (e.g. regression coefficient) AND variation (e.g. standard deviation) or associated estimates of uncertainty (e.g. confidence intervals)
☐	☒	For null hypothesis testing, the test statistic (e.g. *F*, *t*, *r*) with confidence intervals, effect sizes, degrees of freedom and *P* value noted<br>*Give P values as exact values whenever suitable.*
☒	☐	For Bayesian analysis, information on the choice of priors and Markov chain Monte Carlo settings
☐	☒	For hierarchical and complex designs, identification of the appropriate level for tests and full reporting of outcomes
☒	☐	Estimates of effect sizes (e.g. Cohen's *d*, Pearson's *r*), indicating how they were calculated

Our web collection on statistics for biologists contains articles on many of the points above.

Software and code

Policy information about availability of computer code

Data collection	Imaris 9.5.1 (Oxford Instruments, UK)<br>ImageJ 1.49 (National Institutes of Health, MD, USA)
Data analysis	GraphPad Prism 8.0.1 (San Diego, CA, USA)

For manuscripts utilizing custom algorithms or software that are central to the research but not yet described in published literature, software must be made available to editors and reviewers. We strongly encourage code deposition in a community repository (e.g. GitHub). See the Nature Portfolio guidelines for submitting code & software for further information.

Data

Policy information about availability of data

All manuscripts must include a data availability statement. This statement should provide the following information, where applicable:

- Accession codes, unique identifiers, or web links for publicly available datasets
- A description of any restrictions on data availability
- For clinical datasets or third party data, please ensure that the statement adheres to our policy

The authors declare that all data supporting the findings of this study are available within the article and its supplementary information files or from the corresponding author on reasonable request.

March 2021

Human research participants

Policy information about studies involving human research participants and Sex and Gender in Research.

Reporting on sex and gender	*Use the terms sex (biological attribute) and gender (shaped by social and cultural circumstances) carefully in order to avoid confusing both terms. Indicate if findings apply to only one sex or gender; describe whether sex and gender were considered in study design whether sex and/or gender was determined based on self-reporting or assigned and methods used. Provide in the source data disaggregated sex and gender data where this information has been collected, and consent has been obtained for sharing of individual-level data; provide overall numbers in this Reporting Summary. Please state if this information has not been collected. Report sex- and gender-based analyses where performed, justify reasons for lack of sex- and gender-based analysis.*
Population characteristics	*Describe the covariate-relevant population characteristics of the human research participants (e.g. age, genotypic information, past and current diagnosis and treatment categories). If you filled out the behavioural & social sciences study design questions and have nothing to add here, write "See above."*
Recruitment	*Describe how participants were recruited. Outline any potential self-selection bias or other biases that may be present and how these are likely to impact results.*
Ethics oversight	*Identify the organization(s) that approved the study protocol.*

Note that full information on the approval of the study protocol must also be provided in the manuscript.

Field-specific reporting

Please select the one below that is the best fit for your research. If you are not sure, read the appropriate sections before making your selection.

☒ Life sciences ☐ Behavioural & social sciences ☐ Ecological, evolutionary & environmental sciences

For a reference copy of the document with all sections, see nature.com/documents/nr-reporting-summary-flat.pdf

Life sciences study design

All studies must disclose on these points even when the disclosure is negative.

Sample size	The minimum sample size for the mouse islet experiments is 3. Because the large difference between groups, it is sufficient for drawing conclusions. As laboratory zebrafish is not inbred, the standard deviation of alpha cell number is up to 15% within a group. The difference between control and experimental groups is about 30% on average. Power calculation at an alpha=0.05, beta=0.1, Power=0.9 indicated a sample size of at least 4.
Data exclusions	No data were excluded from the analyses.
Replication	The experiments in this study were replicated by at least two individual investigators, and some key data were generated based on a double determination by pharmacological methods and genetic methods. We confirmed that all the attempts at replication were successful.
Randomization	The experimental zebrafish and mice were allocated randomly into each group in this study.
Blinding	The investigators were blinded to group allocation during data collection and analysis.

Reporting for specific materials, systems and methods

We require information from authors about some types of materials, experimental systems and methods used in many studies. Here, indicate whether each material, system or method listed is relevant to your study. If you are not sure if a list item applies to your research, read the appropriate section before selecting a response.

Materials & experimental systems

n/a	Involved in the study
☐	☒ Antibodies
☒	☐ Eukaryotic cell lines
☒	☐ Palaeontology and archaeology
☐	☒ Animals and other organisms
☒	☐ Clinical data
☒	☐ Dual use research of concern

Methods

n/a	Involved in the study
☒	☐ ChIP-seq
☒	☐ Flow cytometry
☒	☐ MRI-based neuroimaging

Antibodies

Antibodies used

Primary antibodies: Anti-GFP (For mouse, 1:300, Abcam, ab13970, Chicken), Anti-pS6 (Ser240/244) (For zebrafish, 1:300, Cell signaling technology, 5364, Rabbit), Anti-HA-probe (For zebrafish, 1:200, Santa Cruz Biotechnology, sc-805, Rabbit), Anti-pERK1/2 (Thr202/Tyr204) (For zebrafish, 1:150, Cell signaling technology, 4370, Rabbit), Anti-Glucagon (For mouse,1:200, Cell signaling technology, 2760, Rabbit) and Anti-Ki67 (For mouse, 1:150, Abcam, ab15580, Rabbit).

Secondary antibodies: Alexa Fluor 488 (1:1,000, Thermo Fisher Scientific, A-11039, Goat anti-Chicken), Alexa Fluor 488 (1:1,000, Thermo Fisher Scientific, A-11001, Goat anti-Mouse), Alexa Fluor 568 (1:1,000, Thermo Fisher Scientific, A-11011, Goat anti-Rabbit) or Alexa Fluor 647 (1:1,000, Thermo Fisher Scientific, A-21235, Goat anti-Mouse)

Validation

Primary antibodies:
Anti-GFP (For mouse, 1:300, Abcam, ab13970, Chicken):This antibody was verified by relative expression to ensure that the antibody binds to the antigen stated.
Anti-pS6 (Ser240/244) (For zebrafish, 1:300, Cell signaling technology, 5364, Rabbit): Supported by supplementary Figure 2a-c in the manuscript.
Anti-HA-probe (For zebrafish, 1:200, Santa Cruz Biotechnology, sc-805, Rabbit): Supported by a published article: Wang, P. et al. Macrophage achieves self-protection against oxidative stress-induced ageing through the Mst-Nrf2 axis. Nat. Commun. 10, 755 (2019).
Anti-pERK1/2 (Thr202/Tyr204) (For zebrafish, 1:150, Cell signaling technology, 4370, Rabbit): Supported by a published article: Chou, Y. et al. Ribose-5-phosphate isomerase A overexpression promotes liver cancer development in transgenic zebrafish via activation of ERK and β-catenin pathways. Carcinogenesis. 40, 461-473 (2019).
Anti-Glucagon (For mouse,1:200, Cell signaling technology, 2760, Rabbit): Supported by a published article: Dean, E. D. et al. Interrupted glucagon signaling reveals hepatic alpha cell axis and role for L-Glutamine in alpha cell proliferation. Cell Metab. 25, 1362-1373 (2017).
Anti-Ki67 (For mouse, 1:150, Abcam, ab15580, Rabbit): Supported by a published article: Dean, E. D. et al. Interrupted glucagon signaling reveals hepatic alpha cell axis and role for L-Glutamine in alpha cell proliferation. Cell Metab. 25, 1362-1373 (2017).

Secondary antibodies:
Alexa Fluor 488 (1:1,000, Thermo Fisher Scientific, A-11039, Goat anti-Chicken): Supported by a published article: Bobkov, O. M. G. et al. Spt6 is a maintenance factor for centromeric CENP-A. Nat. Commun. 11, 2919 (2020).
Alexa Fluor 488 (1:1,000, Thermo Fisher Scientific, A-11001, Goat anti-Mouse): Supported by a published article: Davis, L. J. et al. Single-cell multiomics reveals the complexity of TGFβ signalling to chromatin in iPSC-derived kidney organoids. Commun. Biol. 5, 1301 (2022).
Alexa Fluor 568 (1:1,000, Thermo Fisher Scientific, A-11011, Goat anti-Rabbit), Supported by a published article: Yan, L. et al. SENP1 prevents steatohepatitis by suppressing RIPK1-driven apoptosis and inflammation. Nat. Commun. 13, 7153 (2022).
Alexa Fluor 647 (1:1,000, Thermo Fisher Scientific, A-21235, Goat anti-Mouse), Supported by a published article: Zhao, L. et al. Structure insights into selective coupling of G protein subtypes by a class B G protein-coupled receptor. Nat. Commun. 13, 6670 (2022).

Animals and other research organisms

Policy information about studies involving animals; ARRIVE guidelines recommended for reporting animal research, and Sex and Gender in Research

Laboratory animals

Mouse:
C57BL/6J and Casrflox/flox, 8-18-week-old male and females. The experimental mice were maintained in a controlled environment (12-h light/dark cycle, 22 ± 1 °C, 60%–70% humidity) with free access to standard chow pellets and water.

Zebrafish (all the lines are in the Tg(gcga:EGFP) background:
Tg(gcga:MTORL1460P, cryaa:tagRFP), 5-dpf mixed-gender larvae and 2-3-month-old males and females;
Tg(gcga:RhebS16H, cryaa:YFP), 5-dpf mixed-gender larvae and 2-3-month-old males and females;
gcgra-/-;gcgrb-/-, 5-dpf mixed-gender larvae;
casr-/-, 5-10-dpf mixed-gender larvae or 2-3-month-old males and females;
casr-/-;gcgra-/-;gcgrb-/-, 5-dpf mixed-gender larvae or 2-3-month-old males and females;
Tg(gcga:CaSR, cryaa:tagRFP);casr-/-;gcgra-/-;gcgrb-/-, 5-dpf mixed-gender larvae;
Tg(gcga:hM3Dq, cryaa:tagRFP), 5-dpf mixed-gender larvae;
Tg(gcga:hM3Dq, cryaa:tagRFP);casr-/-;gcgra-/-;gcgrb-/-, 5-dpf mixed-gender larvae;
Tg(gcga:hM3Dq, cryaa:tagRFP);gcgra-/-;gcgrb-/-, 5-dpf mixed-gender larvae;
Tg(gcga:hM4Di, cryaa:tagRFP), 5-dpf mixed-gender larvae;

	Tg(gcga:hM4Di, cryaa:tagRFP);casr-/-;gcgra-/-;gcgrb-/-, 5-dpf mixed-gender larvae;<br>Tg(gcga:hM4Di, cryaa:tagRFP);gcgra-/-;gcgrb-/-, 5-dpf mixed-gender larvae;<br>Tg(gcga:RhebS16H, cryaa:YFP);Tg(gcga:hM3Dq, cryaa:tagRFP), 5-dpf mixed-gender larvae.
Wild animals	This study did not involve wild animals.
Reporting on sex	This study used mixed-gender zebrafish larvae and male and female mice.
Field-collected samples	This study did not involve samples collected from the field.
Ethics oversight	The animals were handled in compliance with guidelines approved by the Vanderbilt University Institutional Animal Care and Use Committee protocols (protocol no. M1700143-01 and M2000070-00).

Note that full information on the approval of the study protocol must also be provided in the manuscript.
